# Proteomic analysis of filaggrin deficiency identifies molecular signatures characteristic of atopic eczema

**DOI:** 10.1016/j.jaci.2017.01.039

**Published:** 2017-11

**Authors:** Martina S. Elias, Heather A. Long, Carla F. Newman, Paul A. Wilson, Andrew West, Paul J. McGill, Keith C. Wu, Michael J. Donaldson, Nick J. Reynolds

**Affiliations:** a Dermatological Sciences, Institute of Cellular Medicine, Newcastle University, Newcastle upon Tyne, United Kingdom; b GlaxoSmithKline R&D, Stevenage, United Kingdom; c Stiefel, a GlaxoSmithKline company, Stevenage, United Kingdom; d Department of Dermatology, Royal Victoria Infirmary, Newcastle upon Tyne, United Kingdom

**Keywords:** Atopic eczema, dermatitis, skin, proteomic, filaggrin, kallikrein-7, cyclophilin A, AE, Atopic eczema, ANXA3, Annexin A3, CFL1, Cofilin-1, CTSV, Cathepsin V, eIF, Eukaryotic initiation factor, FLG, Filaggrin, IPA, Ingenuity Pathway Analysis, KLK7, Kallikrein-7, LSE, Living skin equivalent, mTOR, Mammalian target of rapamycin, PPIA, Cyclophilin A, RT-qPCR, Quantitative RT-PCR, shNT, Nontargeting control short hairpin RNA, shFLG, Filaggrin knockdown short hairpin RNA, TXN, Thioredoxin, VIM, Vimentin

## Abstract

**Background:**

Atopic eczema (AE) is characterized by skin barrier and immune dysfunction. Null mutations in filaggrin (FLG), a key epidermal barrier protein, strongly predispose to AE; however, the precise role of FLG deficiency in AE pathogenesis remains incompletely understood.

**Objectives:**

We sought to identify global proteomic changes downstream of FLG deficiency in human epidermal living skin–equivalent (LSE) models and validate findings in skin of patients with AE.

**Methods:**

Differentially expressed proteins from paired control (nontargeting control short hairpin RNA [shNT]) and FLG knockdown (FLG knockdown short hairpin RNA [shFLG]) LSEs were identified by means of proteomic analysis (liquid chromatography–mass spectrometry) and Ingenuity Pathway Analysis. Expression of key targets was validated in independent LSE samples (quantitative RT-PCR and Western blotting) and in normal and AE skin biopsy specimens (immunofluorescence).

**Results:**

Proteomic analysis identified 17 (*P* ≤ .05) differentially expressed proteins after FLG knockdown, including kallikrein-7 (KLK7; 2.2-fold), cyclophilin A (PPIA; 0.9-fold), and cofilin-1 (CFL1, 1.3-fold). Differential protein expression was confirmed in shNT/shFLG LSEs; however, only KLK7 was transcriptionally dysregulated. Molecular pathways overrepresented after FLG knockdown included inflammation, protease activity, cell structure, and stress. Furthermore, KLK7 (1.8-fold) and PPIA (0.65-fold) proteins were differentially expressed in lesional biopsy specimens from patients with AE relative to normal skin.

**Conclusions:**

For the first time, we show that loss of FLG in the absence of inflammation is sufficient to alter the expression level of proteins relevant to the pathogenesis of AE. These include proteins regulating inflammatory, proteolytic, and cytoskeletal functions. We identify PPIA as a novel protein with levels that are decreased in clinically active AE skin and show that the characteristic upregulation of KLK7 expression in patients with AE occurs downstream of FLG loss. Importantly, we highlight disconnect between the epidermal proteome and transcriptome, emphasizing the utility of global proteomic studies.

## Graphical abstract

**Figure:**
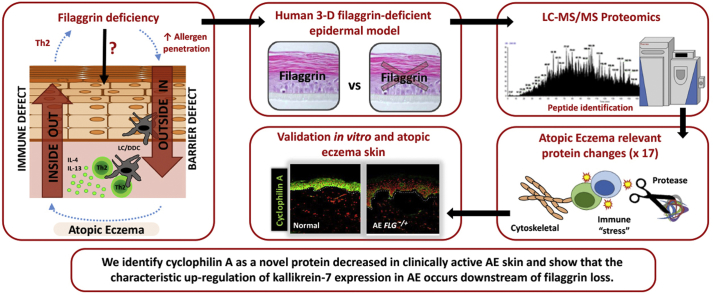

Filaggrin (FLG) is a major constituent of the epidermal barrier, contributing to its structure, function, and hydration.^1^ Both single- and double-allele loss-of-function mutations in *FLG* strongly predispose to the development of atopic eczema (AE) and secondary atopic conditions, including asthma and allergic rhinitis.^2^, ^3^, ^4^ To date, more than 40 different population-specific *FLG* mutations have been identified, each resulting in a truncated profilaggrin gene product, which is not processed into functional FLG monomers.^3^ Up to 50% of cases of moderate-to-severe AE in northern Europe can be attributed, at least in part, to an *FLG*-null mutation,^5^, ^6^ representing the strongest and most consistent genetic risk factors identified for AE to date (overall odds ratio, 3.12-4.78).^6^, ^7^ Furthermore, *FLG* is polymorphic, with common allelic variants encoding a profilaggrin molecule composed of 10, 11, or 12 FLG monomeric repeats. Each additional FLG repeat confers a reduced risk of AE by a factor of 0.88, suggesting that even small increases in *FLG* expression might be therapeutically beneficial.^8^

Both the flaky tail mouse (which carries a natural mutation virtually ablating all FLG expression^9^) and FLG knockout mice exhibit enhanced percutaneous antigen transfer and allergen/irritant-induced AE-like inflammatory responses.^10^, ^11^ These models support a primary role for FLG and highlight the importance of a functional cutaneous barrier in AE disease pathogenesis.

Recent cohort studies have examined global gene expression changes in patients with AE stratified by FLG genotype.^12^, ^13^ Although interesting, their interpretation with regard to the direct role of FLG in AE pathogenesis is confounded by the complex interplay between the epidermal barrier, immune system, and environment.^14^ For example, loss of FLG is also observed in patients with *FLG* wild-type AE, likely because of its extrinsic downregulation by the atopic T_H_2-polarized microenvironment.^15^ Moreover, murine models are additionally limited both by secondary mutations, notably null mutations in *TMEM79/Matt* (Mattrin) in the flaky tail mouse,^16^ and interspecies differences. The human and murine *FLG* sequences lack homology,^17^ and murine models do not display the heterozygote phenotype typical of most patients with AE.^10^, ^11^ Consequently, the precise molecular changes occurring directly as a result of FLG loss are relatively unknown.

A useful alternative tool for the study of epidermal biology is the 3-dimensional living skin–equivalent (LSE) model derived exclusively from primary human keratinocytes. Although by definition these simplified models do not recapitulate the complexity of human AE skin, they enable the study of epidermal biology in the absence of confounding inflammatory cells.^18^ Furthermore, they enable the effect of *FLG* gene silencing to be analyzed in a pairwise manner on a homogeneous genetic background.

Previous LSE studies have largely focused on understanding the ultrastructural and functional consequence of FLG deficiency.^19^, ^20^, ^21^ In many regards these mirror changes observed in AE skin, supporting their utility as a disease model. However, to the best of our knowledge, no systematic transcriptomic or proteomic analysis has been performed after *FLG* knockdown in human epidermis. The aim of this study was to use FLG knockdown LSE models to investigate the global molecular consequences resulting directly from *FLG* deficiency. A proteomics-based approach was used because protein changes largely represent the functional end point of cell signaling, and recent studies have identified an important disconnect between the transcriptome and proteome, suggesting that significant posttranscriptional regulation occurs.^22^

Notably, for the first time, we have identified 17 proteins that are significantly differentially expressed after FLG knockdown in LSE cultures. Bioinformatic analysis was used to categorize and align these to putative regulatory networks and showed that loss of FLG alone is sufficient to induce protein changes relevant to the pathogenesis of AE. Specifically, pathways relating to protease activity, inflammation, cell structure, and stress were overrepresented after FLG knockdown. The expression profile of key targets, namely the AE-relevant protease kallikrein-7 (KLK7), the novel AE immune modulator cyclophilin A (PPIA), and the actin-binding protein cofilin-1 (CFL1), were replicated first in independent FLG knockdown LSEs and then further characterized in skin biopsy specimens from patients with AE.

## Methods

### Primary keratinocyte culture

Normal human epidermal keratinocytes extracted from surplus foreskin tissue obtained after informed biobank consent were cultured in low-calcium (0.06 mmol/L) EpiLife supplemented with 1% human keratinocyte growth supplement (Life Technologies, Paisley, United Kingdom), as previously described.^23^ All donor subjects had no history of AE.

### Lentivirus (short hairpin RNA) production and transduction of primary keratinocytes

Lentivirus production from GIPZ lentiviral shRNAmir vectors (Open Biosystems, Lafayette, Colo) targeting FLG (filaggrin knockdown short hairpin RNA [shFLG], V3LHS_369921) or a nontargeting control sequence (nontargeting control short hairpin RNA [shNT], RHS4346) and keratinocyte transduction were performed, as previously described.^24^ Further details are provided in the Methods section in this article's Online Repository at www.jacionline.org.

### LSE culture and protein extraction

LSEs were generated from independent biological donors (n = 10 for proteomic analysis and a further n = 3-8 for validation experiments) and proteins extracted in 8 mol/L urea lysis buffer, as previously described.^24^ Further details are provided in this article's Online Repository.

### Patient samples

Patients with AE (as diagnosed per the United Kingdom–modified Hanifin and Rajka criteria^25^) were recruited from a tertiary referral dermatology clinic. Patients older than 18 years not receiving systemic immune-modifying drugs were included in the study after written informed consent and a 2-week washout period from potent topical steroids. Five-millimeter punch biopsy specimens were taken from both lesional and nonlesional skin after achievement of local anesthesia. After obtaining informed consent, normal (control) skin was collected after plastic surgery or excision of benign cutaneous lesions. A blood sample was also collected from all patients and control subjects for *FLG* mutational analysis (further details provided in this article's Online Repository). All control subjects reported in the study had no history of AE and were *FLG* wild-type. This study was approved by the Regional Research Ethics Committee and was conducted according to the Declaration of Helsinki principles.

### Immunoblotting and densitometry

Immunoblotting was performed by using standard protocols, as previously described.^24^ Primary antibody conditions were as follows: FLG clone 15C10 (1:200; NCL-FLG; Leica Biosystems, Newcastle upon Tyne, United Kingdom), CFL1 (1:500; PA5-27627, Thermo Scientific, Waltham, Mass), KLK7 (1:1000; AF2624; R&D Systems, Minneapolis, Minn), PPIA (1:300; 39-1100; Life Technologies), and glyceraldehyde-3-phosphate dehydrogenase (1:10,000; #2118; Cell Signaling Technology, Danvers, Mass). Further details are provided in this article's Online Repository.

### Proteomic and data analysis

Proteins were prepared by means of in-solution or in-gel digestion and subjected to liquid chromatography/mass spectrometry proteomic analysis. Data analysis was performed with Proteome Discoverer (Thermo Fisher Scientific) and Scaffold (Proteome Software, Portland, Ore). Protein identifications were accepted if they could be established at greater than 99.9% probability and contained at least 2 identified peptides. Further details are provided in this article's Online Repository.

### Immunofluorescence

OCT-embedded LSE and skin cryosections (4 μm) were fixed and stained, as previously described.^24^ Primary antibody conditions were as follows: FLG (Acetone fix, 1:100; MS-449-P1; Thermo Scientific), CFL1 (paraformaldehyde fix, 1:300; PA5-27627; Thermo Scientific), KLK7 (Methanol fix, 1:250; AF2624; R&D Systems), and PPIA (paraformaldehyde fix, 1:500; 39-1100; Life Technologies). Quantification was performed with ImageJ software (National Institutes of Health, Bethesda, Md). Further details are provided in this article's Online Repository.

### Quantitative RT-PCR

Quantitative RT-PCR (RT-qPCR) was performed, as described by Forrester et al,^24^ with exon-spanning probe-based assays: FLG (Hs.PT.53a.23095403, FAM/TAMRA; Integrated DNA Technologies, Coralville, Iowa), PPIA (forward: ATGCTGGACCCAACACAAAT; reverse: TCTTTCACTTTGCCAAACACC; UPL PROBE#48 FAM/NFQ; Roche, Mannheim, Germany), CFL1 (forward: GTGCCCTCTCCTTTTCGTTT; reverse: TTGAACACCTTGATGACACCAT; UPL PROBE#5 FAM/NFQ; Roche), and KLK7 (Hs.PT.58.39819237, FAM/TAMRA; Integrated DNA Technologies). *18S* was used as a housekeeping gene for normalization purposes.^26^ Further details are provided in this article's Online Repository.

### Statistical analysis

Unless otherwise stated in the figure legends, data points represent means ± 95% CIs. Statistical analysis was performed with Prism 5 software (GraphPad Software, La Jolla, Calif). Further details are provided in this article's Online Repository.

## Results

### Establishment of an FLG-deficient human LSE model

**Fig 1:**
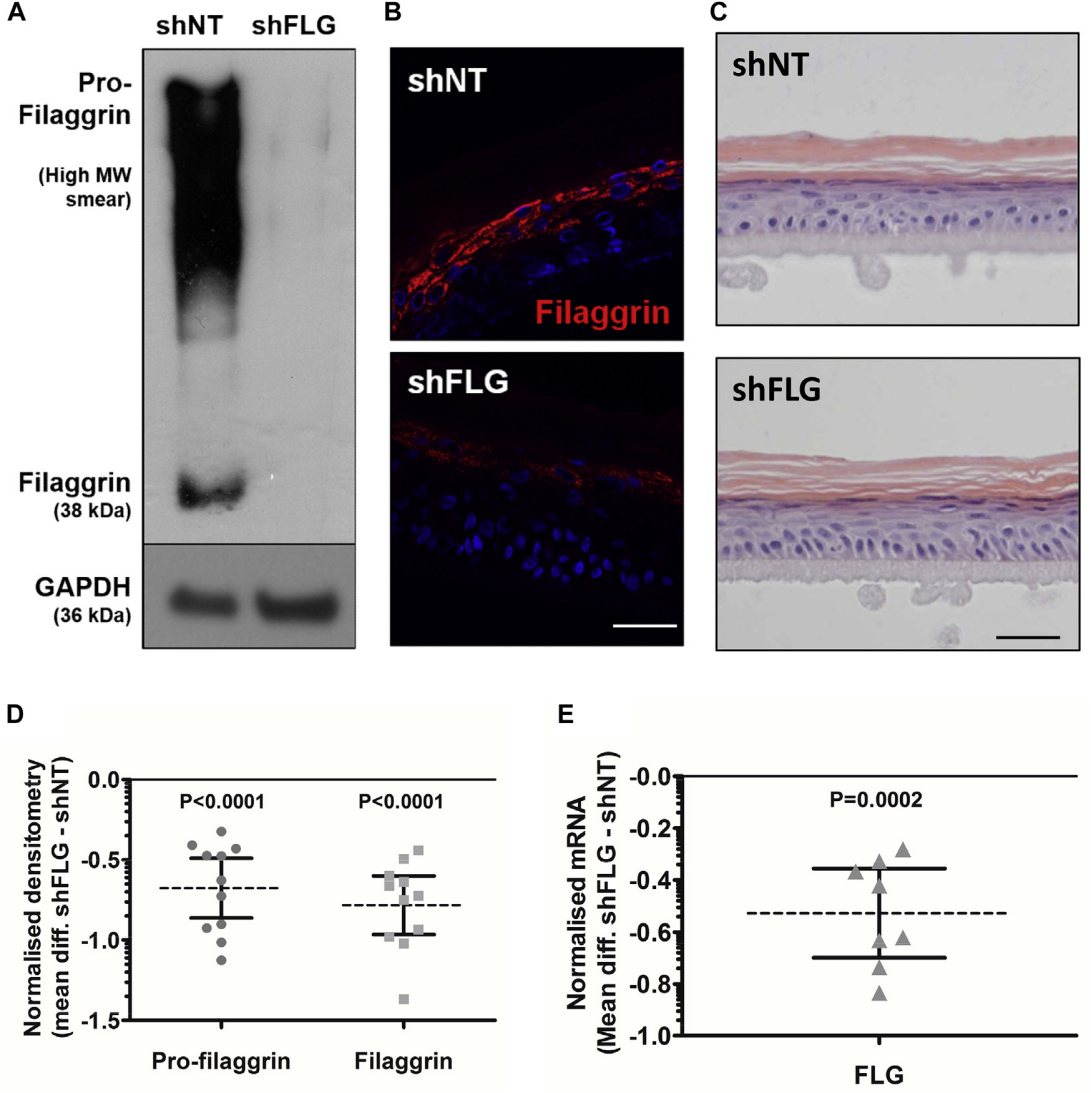
Generation of shFLG and shNT LSE models. **A** and **B,** Representative FLG immunoblot (n = 11; Fig 1, *A*) and immunofluorescence (−150 μm, n = 3; Fig 1, *B*). *MW*, Molecular weight. **C,** Hematoxylin and eosin–stained LSE morphology (−100 μm, n = 10). **D** and **E,** Normalized FLG densitometry of immunoblots (n = 11; Fig 1, *D*) and TaqMan mRNA expression levels (n = 8; Fig 1, *E*). Scatter plots of log_10_-transformed data (means ± 95% CIs).

**Fig E1:**
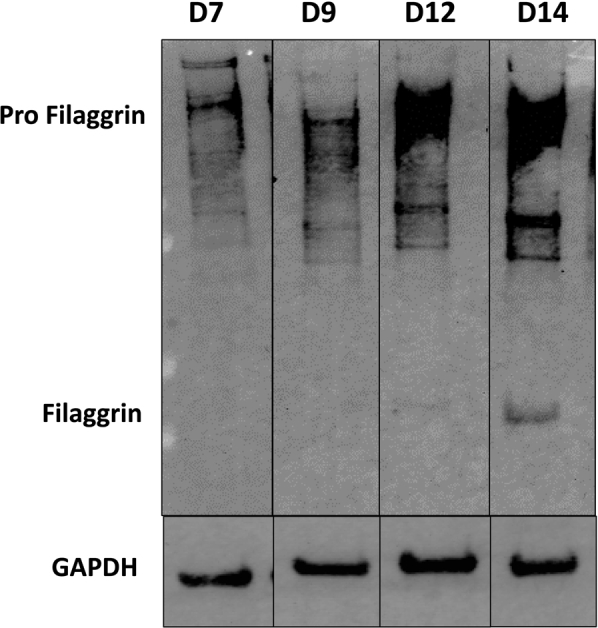


**Fig E2:**
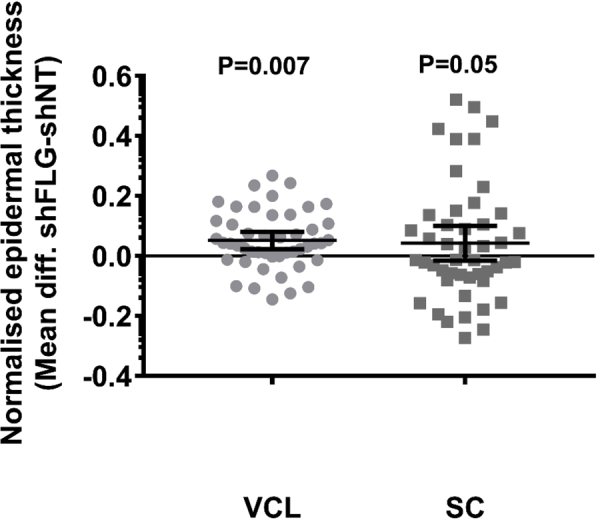


Reliable and reproducible knockdown of FLG expression in primary keratinocytes was achieved by using a second-generation pGIPZ lentiviral shRNAmir construct targeting the *FLG* mRNA sequence (shFLG). A nontargeting shRNAmir construct was used as a control (shNT). LSEs were established from transduced keratinocytes, and protein and mRNA knockdown was assessed at day 14 of culture, the time point at which LSEs are fully differentiated (Fig 1 and see Fig E1 in this article's Online Repository at www.jacionline.org). Densitometry of immunoblots confirmed significant knockdown in both profilaggrin (75% ± 4.1%, *P* ≤ .0001) and FLG (79% ± 3.1%, *P* ≤ .0001) proteins (Fig 1, *A*, *B*, and *D*). RT-qPCR confirmed a significant reduction in *FLG* mRNA expression (69% ± 4.9%, *P* ≤ .0001; Fig 1, *E*). Histologic examination of LSE sections showed no overt phenotypic changes between nonvirally modified LSEs and the shNT control LSEs (data not shown) or between the shNT and shFLG LSEs (Fig 1, *C*). However, quantification with ImageJ analysis software identified a small but significant increase in epidermal thickness after FLG knockdown compared with the shNT control. As such, both a thicker viable cell layer (1.14 ± 0.03-fold, *P* = .0007) and stratum corneum (1.13 ± 0.06-fold, *P* = .05) were observed (see Fig E2 in this article's Online Repository at www.jacionline.org).

### Seventeen proteins were significantly differentially regulated after FLG knockdown in human epidermal skin equivalent

**Fig 2:**
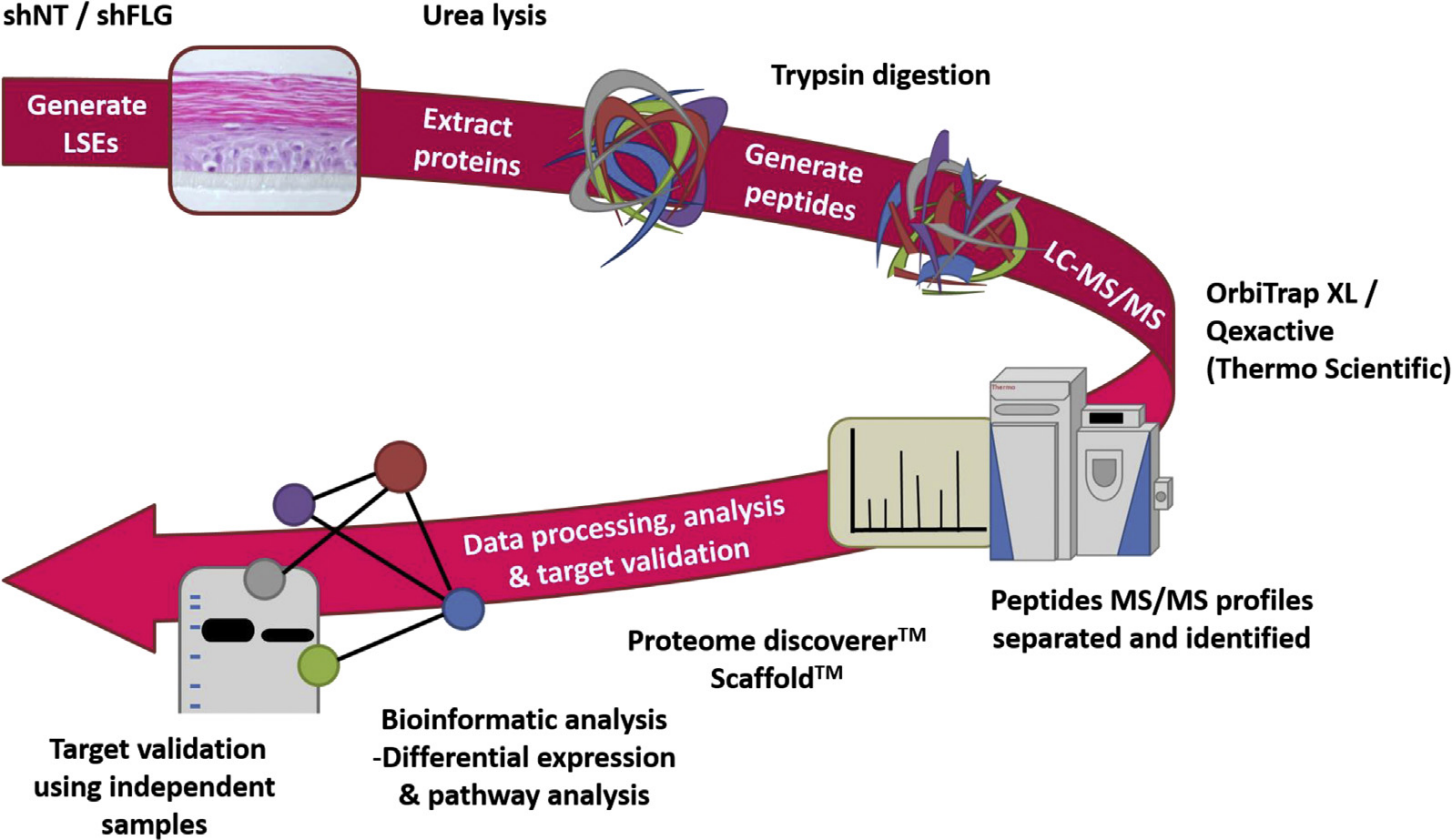
Overview of the open proteomic workflow. Peptides were extracted from shNT and shFLG LSEs and separated by means of liquid chromatography mass spectrometry (*LC-MS* and *LC-MS/MS*). Proteins were identified from MS/MS spectra, and unweighted spectrum counts were used to determine relative expression levels. Selected targets were independently validated.

**Fig E3:**
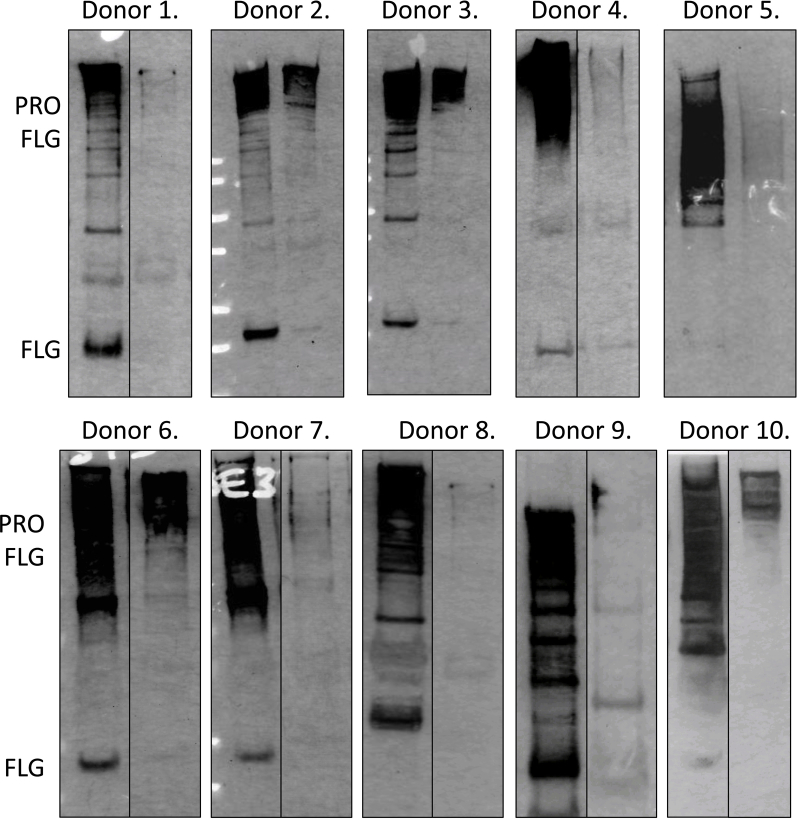


**Fig E4:**
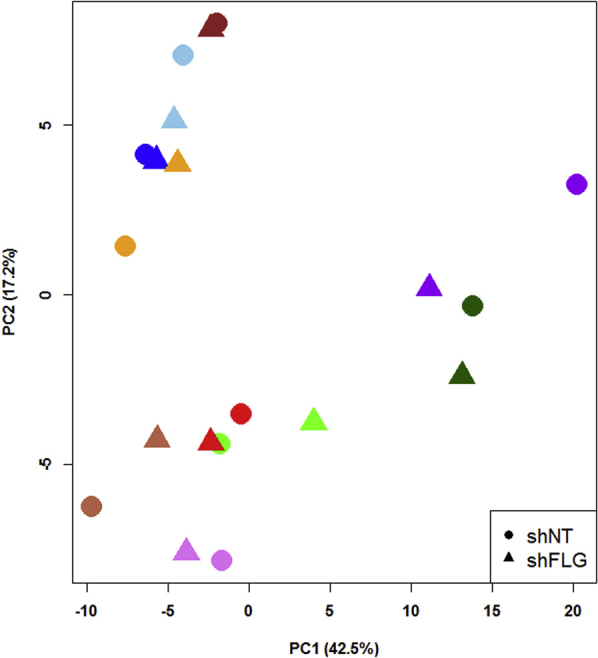


**Fig E5:**
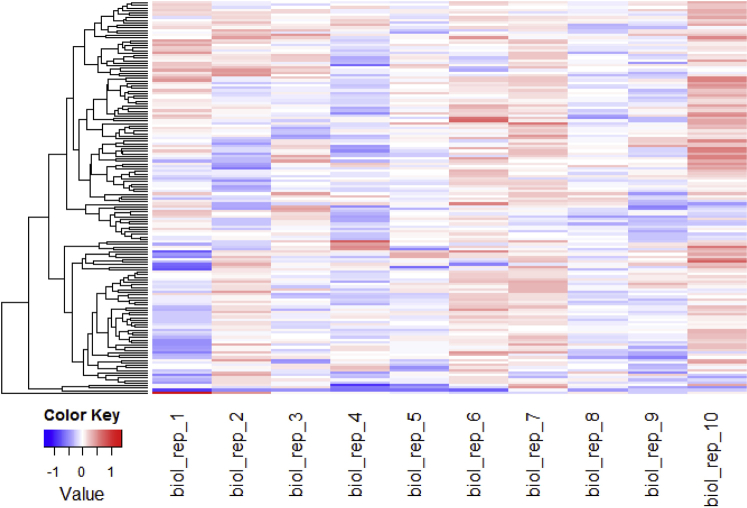


The soluble protein fraction from paired shNT and shFLG LSE samples generated from 10 independent biological human donors displaying normal patterns of baseline FLG expression (see Fig E3 in this article's Online Repository at www.jacionline.org) were subject to an open proteomic workflow (Fig 2, *A*). One thousand six hundred forty unique proteins were identified across all samples, with an average of 634 proteins identified per LSE. Of these, 367 proteins were identified in at least 75% of the shNT and shFLG paired samples (see Table E1 in this article's Online Repository at www.jacionline.org). The unweighted spectrum counts from this subset of proteins were used in subsequent analysis. Principal component analysis reported clustering by biological donor and not FLG status (see Fig E4 in this article's Online Repository at www.jacionline.org). To account for the prominent donor effect, log_10_ differences between shNT and shFLG conditions were calculated for each biological donor (ie, a paired design), and log_10_ estimated differences in expression profile were used for input for statistical analysis. A summary of the differential expression profile for each biological donor is represented as a heat map (see Fig E5 in this article's Online Repository at www.jacionline.org). Overlaid dendrogram plots depict clustering between protein response profiles.

**Fig 3:**
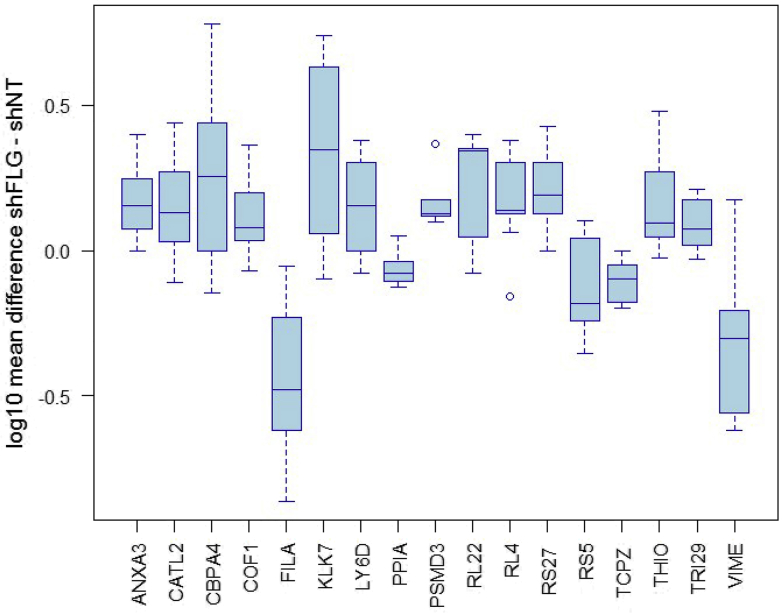
Box plot showing differentially expressed proteins after FLG knockdown. Log_10_ mean protein differences (shFLG − shNT) were calculated independently for each biological replicate and collated for statistical analysis (n = 10). Displayed proteins were differentially regulated. *P* ≤ .05, 1-sample *t* test. Values are medians and interquartile ranges.

Notwithstanding the prominent intradonor variability, statistical analysis of the combined data set identified 17 differentially regulated proteins (*P* ≤ .05; Fig 3 and see Table E2 in this article's Online Repository at www.jacionline.org). Positive/negative mean differences represent upregulated/downregulated proteins after FLG knockdown, respectively. Differentially expressed proteins broadly relate to a range of functional classes, including proteases (KLK7, cathepsin V [CTSV], and carboxypeptidase A4 [CPA4]), inflammatory mediators (annexin A3 [ANXA3], lymphocyte antigen 6D [LY6D], PPIA, and thioredoxin [TXN]), and cytoskeletal interactors (FLG, vimentin [VIM], CFL1, T-complex protein 1 subunit zeta [CCT6A], and tripartite motif-containing protein 29 [TRIM29]). The most significantly downregulated protein was FLG (*P* = 2.0E-04), internally validating the analysis methodology.

### FLG-deficient LSEs display expression changes consistent with inflammatory dermatoses

**Fig 4:**
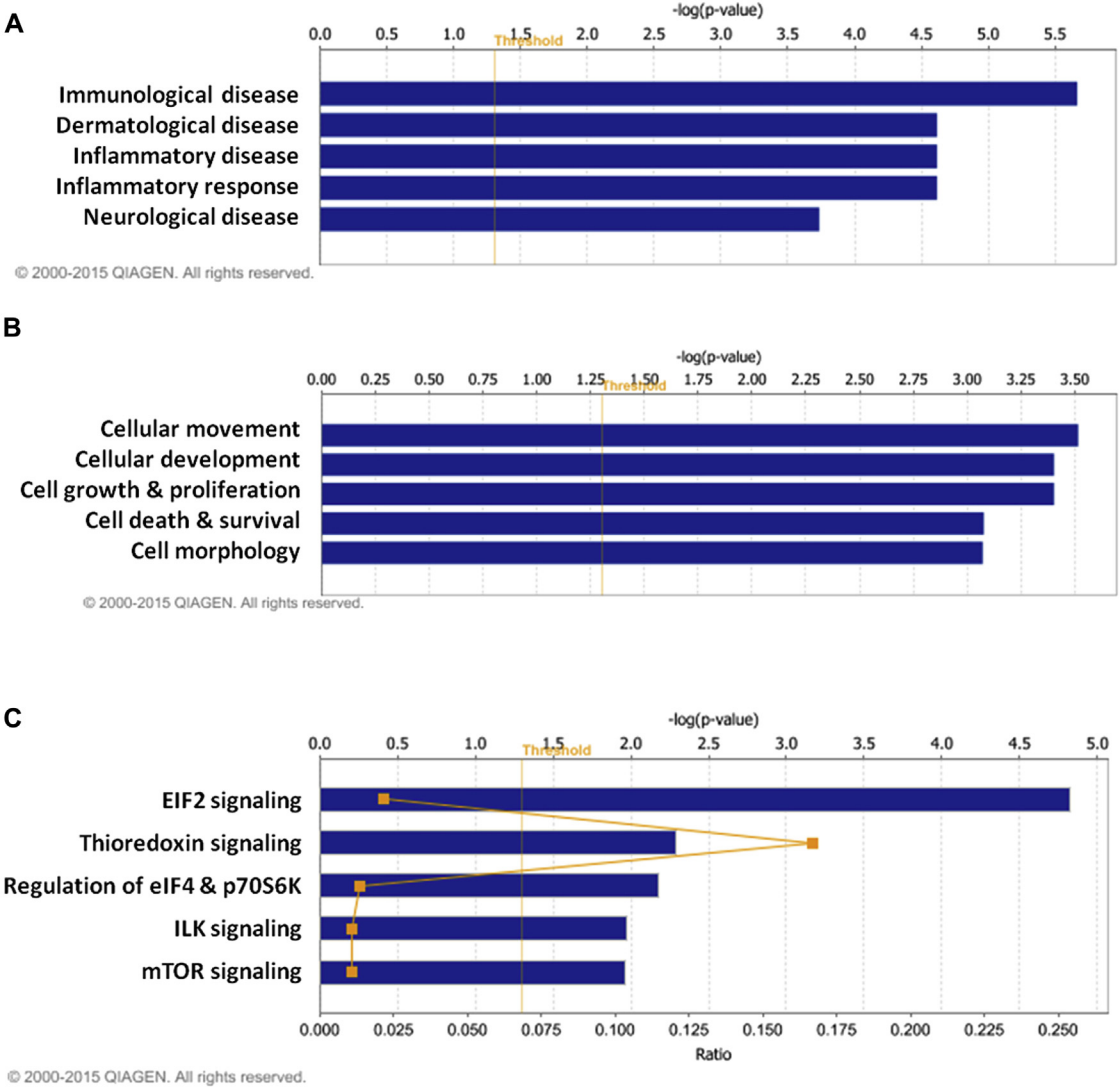
Summary of IPA core functional analysis. Log_10_ mean protein differences (shFLG − shNT) with an adjusted *P* value of less than .05 (threshold) were considered significantly differentially expressed and used as input for IPA. The top 5 “disease and disorders” **(A)**, “molecular and cellular functions” **(B)**, and “canonical pathways” **(C)** altered after FLG knockdown are depicted. The *orange line* depicts the ratio (number of pathway-relevant proteins in the data set divided by the total number of proteins in the pathway).

Ingenuity Pathway Analysis (IPA) identified “immunological disease” (*P* = 2.14E-06 to 3.58E-02, 9 proteins), “dermatological disease” (*P* = 2.42E-05 to 3.99E-02, 6 proteins), and “inflammatory disease” (*P* = 2.42E-05 to 3.66E-03, 7 proteins; Fig 4, *A*, and see Table E3 in this article's Online Repository at www.jacionline.org) as the categories most significantly associated with FLG knockdown. Interestingly, within these categories, the most significantly associated functional term was “allergy” (*P* = 2.15E-06, 6 proteins). A detailed breakdown of the functional terms underlying the top 5 associated disease categories is included in Table E4 in this article's Online Repository at www.jacionline.org.

### FLG knockdown in LSEs leads to enrichment in proteins associated with cellular assembly

Further IPA analysis identified that the “molecular and cellular functions” most significantly overrepresented in our data set were “cellular movement” (*P* = 3.05E-04 to 4.96E-02, 7 proteins), “cellular development” (*P* = 3.94E-04 to 4.47E-02, 7 proteins), and “cell growth and proliferation” (*P* = 3.94E-04 to 4.47E-02, 6 proteins; Fig 4, *B*, and see Table E5 in this article's Online Repository at www.jacionline.org). Significant functional terms included “migration of cells” (*P* = 3.06E-03, 7 proteins) and “differentiation of cells” (*P* = 2.94E-02, 6 proteins). Full details of the functional terms associated with the top 5 cellular function categories are provided in Table E4.

**Fig E6:**
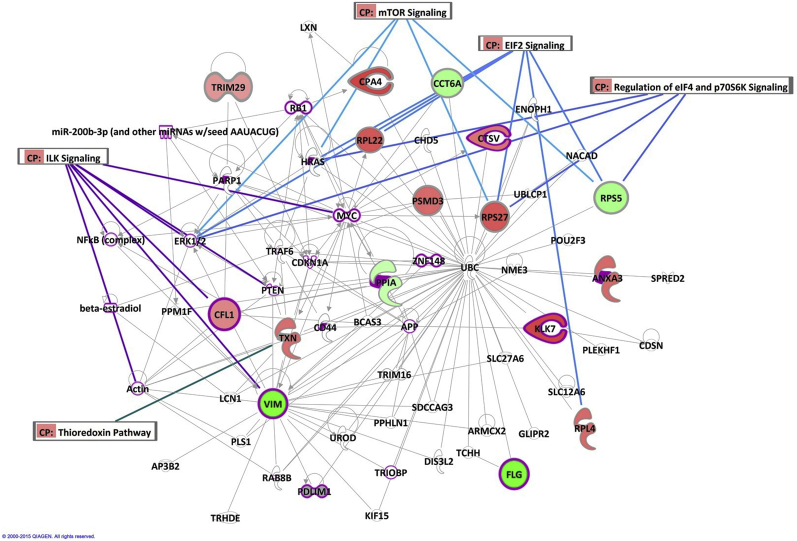


Network analysis returned a single putative network linking all of the differentially regulated proteins with an IPA p-score of 45 (p-score = −log_10_ [*P* value], see Fig E6 in this article's Online Repository at www.jacionline.org). Consistent with the previous analysis, significant functional terms associated with the network included “hyperplasia of epithelial cells” (*P* = 1.02E-06, 7 proteins) and “formation of skin” (*P* = 3.69E-06, 12 proteins). Proteins implicated in dermatological and/or inflammatory disease have been highlighted in pink (see Fig E6).

### Proteins involved in cell stress signaling pathways are overrepresented in LSEs after FLG knockdown

The canonical pathways most significantly associated with the differentially expressed proteins included “EIF2 signaling” (*P* = 1.51E-05, 4 proteins), “regulation of eukaryotic initiation factor (eIF) 4 and p70S6K signaling” (*P* = 1.40E-03, 2 proteins), and “mammalian target of rapamycin (mTOR) signaling” (*P* = 1.08E-02, 2 proteins; Fig 4, *C*, and see Table E6 in this article's Online Repository at www.jacionline.org). The top 5 significantly associated canonical pathways are also overlaid onto the putative molecular network (see Fig E6).

### Confirmation of proteomics results in independent LSEs

A number of differentially expressed proteins were selected for independent validation at the protein and mRNA level in independent shNT and shFLG LSEs derived from biological donors distinct from those used in the original study to confirm the findings from the proteomic analysis. The expression profiles of key representatives from a distinct protein class, such as proteases (KLK7), immunomodulatory functions (PPIA), and cytoskeletal interacting proteins (CFL1), were selected based on their low *P* value, high donor ratio, and relevance to skin morphology/disease.

**Fig 5:**
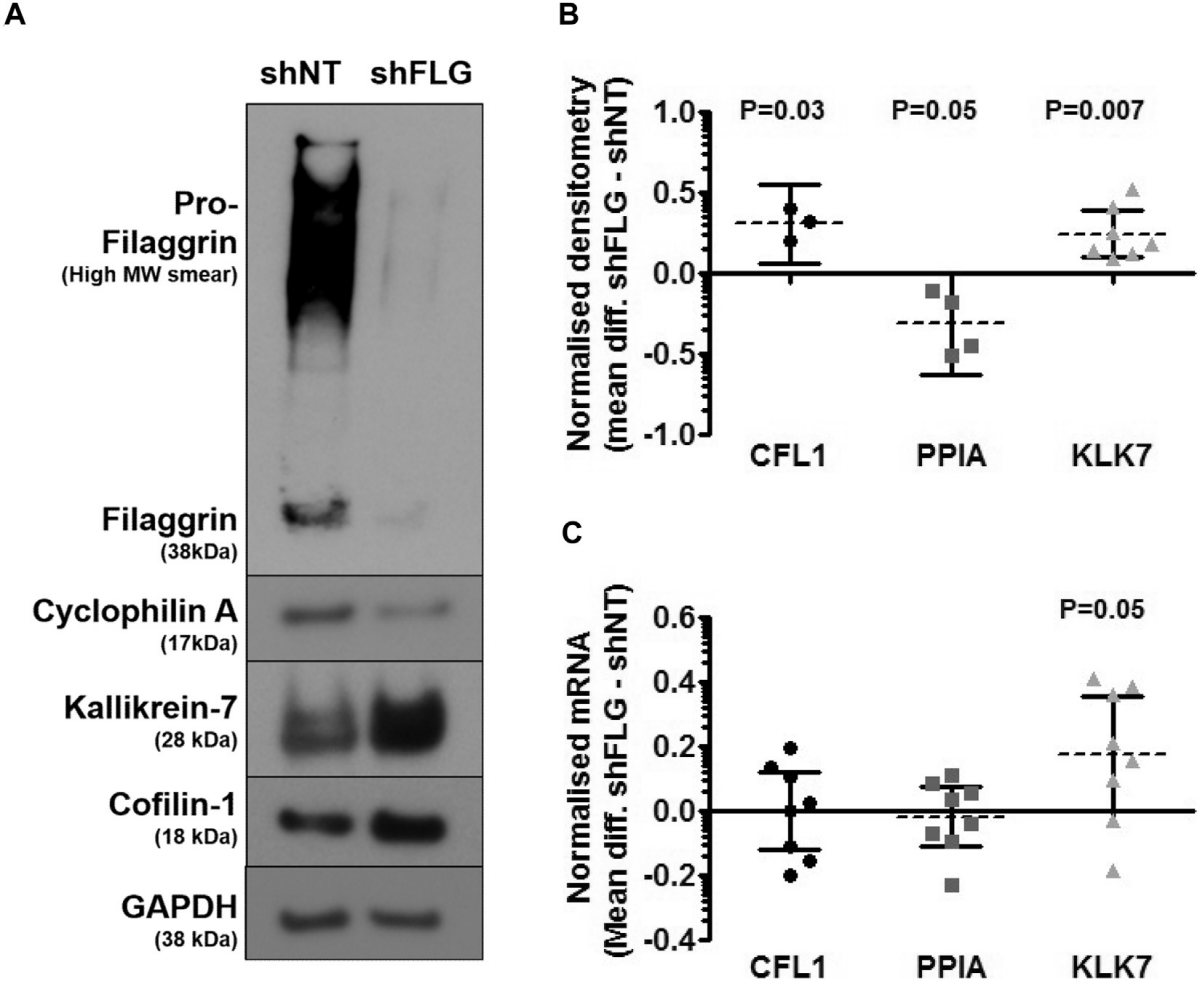
Validation of proteomics findings. Normalized expression levels of CFL1, PPIA, and KLK7 were determined in shNT and shFLG LSEs generated from independent biological samples. **A** and **B,** Representative immunoblots (n ≥ 3; Fig 5, *A*) and associated densitometric quantification (n ≥ 3; Fig 5, *B*). *MW*, Molecular weight. **C,** TaqMan mRNA expression levels (n = 8). Scatter plots of log_10_-transformed data (means ± 95% CIs).

In line with the proteomic results, all 3 proteins were significantly differentially expressed, as assessed by means of immunoblotting and densitometric analysis in the independent LSEs (Fig 5, *A* and *B*). Furthermore, both the relative magnitude and direction of response were preserved: CFL1, 2.1 ± 0.27-fold (*P* = .03), KLK7, 1.89 ± 0.29-fold (*P* = .007), and PPIA, 0.52 ± 0.11-fold (*P* = .05; Fig 5, *A* and *B*).

In contrast to the protein data, there was no significant difference in *CFL1* and *PPIA* mRNA expression levels between shNT and shFLG LSEs, as assessed by using probe-based RT-qPCR assays, underscoring the relevance of a proteomics-based approach. However, *KLK7* mRNA expression was increased in the shFLG condition, which is consistent with the protein data (1.2 ± 0.06-fold, *P* = .05; Fig 5, *C*).

### Replication of FLG knockdown LSE findings in AE skin

Protein expression analysis was performed on human skin from healthy control subjects and patients with AE to determine whether these *in vitro* findings were relevant to the *in vivo* AE disease setting. AE samples were collected from sites of both clinically active eczema (involved skin) and macroscopically normal skin (uninvolved).

**Fig 6:**
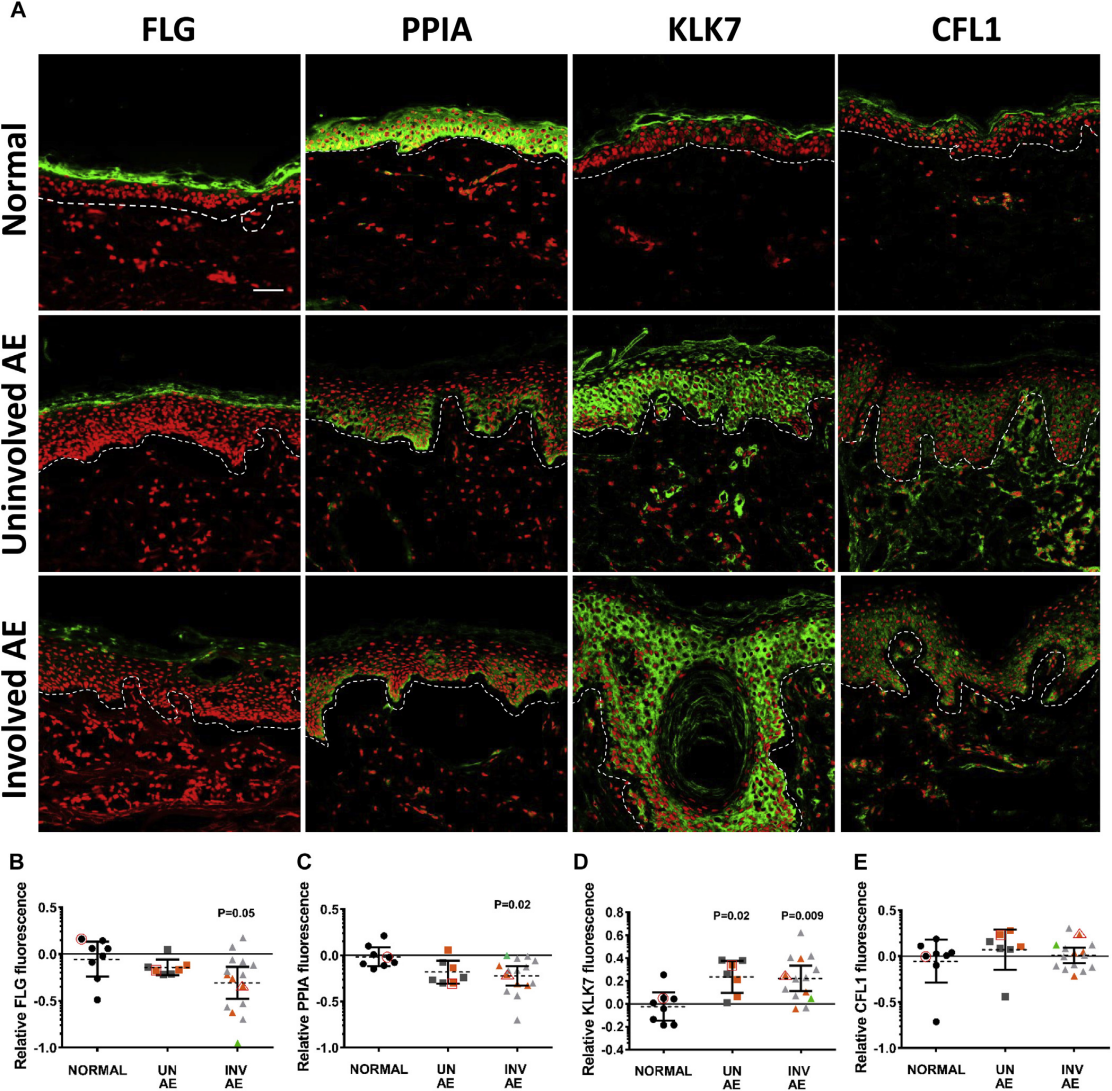
Replication of FLG knockdown LSE findings in AE skin. Representative immunofluorescence images of normal (*FLG* wild-type), uninvolved *(UN AE)*, and involved *(INV AE)* AE skin (n ≥ 3). Green staining depicts FLG, PPIA, KLK7, or CFL1, as indicated. Hoechst 33342 nuclear counterstaining is shown in red (−50 μm). *Dashed lines* represent the epidermal/dermal boundary. **A,** Corresponding quantification values are outlined in red on the bottom scatter plots. **B-E,** Scatter plots of the corresponding protein florescence intensities expressed as function of unit area and on a log_10_ scale (means ± 95% CIs). All subjects are *FLG* wild-type unless indicated in orange (*FLG* heterozygous) or green (*FLG* homozygous/compound heterozygous).

Protein expression of FLG, PPIA, KLK7, and CFL1 was assessed by using a combination of immunofluorescence imaging (Fig 6, *A*) and quantification by means of image analysis (Fig 6, *B-E*). *FLG* mutational status of all control subjects and patients with AE was determined by using next-generation sequencing. All control subjects reported were *FLG* wild-type, and 31% of subjects with AE carried *FLG* mutations (Fig 6, *B-E*, and see Table E7 in this article's Online Repository at www.jacionline.org).

Overall FLG expression was reduced in both uninvolved and involved AE skin compared with that in healthy control subjects, although significance was only observed at sites of clinically active disease (0.6 ± 0.1-fold, *P* ≤ .05; Fig 6, *A* and *B*). FLG expression was particularly reduced in *FLG*-heterozygous subjects and almost absent in the *FLG* double-allele mutant subject (Fig 6, *A* and *B*), and accordingly, a significant correlation between *FLG* genotype (number of expressed alleles) and expression was observed (*r* = 0.48, *P* = .007) across the whole data set.

The protein abundance of PPIA was significantly reduced in involved AE skin compared with control skin (0.65 ± 0.06-fold, *P* ≤ .02), whereas KLK7 expression was significantly increased in both uninvolved (1.8 ± 0.02-fold, *P* ≤ .02) and involved (1.84 ± 0.02-fold, *P* ≤ .009) AE skin. There was a trend toward correlation between FLG and PPIA expression (*r* = 0.31, *P* = .09) and a significant inverse correlation between FLG and KLK7 expression (*r* = −0.38, *P* = .04). However, in neither instance did protein expression stratify or correlate according to *FLG* mutational status (PPIA: *r* = 0.08, *P* = .68; KLK7: *r* = −0.18, *P* = .34; Fig 6, *A-D*). In contrast, the abundance of CFL1 was unchanged between normal and AE skin and did not display any correlation with respective FLG expression (*r* = −0.12, *P* = .51; Fig 6, *A*).

## Discussion

In this study we used an *in vitro* human LSE model to investigate the proteomic changes arising from FLG deficiency *per se* in the absence of any confounding systemic inflammation.^19^ A global proteomics approach and IPA strikingly revealed that shFLG LSEs displayed a disease profile consistent with inflammatory dermatologic disease and an enrichment in proteins associated with cellular assembly. Significant functional terms included “allergy” (increased: ANXA3, CFL1, CTSV, and KLK7; decreased: FLG and PPIA) and “differentiation of cells” (increased: CFL1, CTSV, and RPL22; decreased: FLG, PPIA, and VIM), suggesting that our FLG knockdown model displays many molecular characteristics typical of AE (Fig 4 and see Tables E3-E6). Similar functional annotations, specifically inflammatory and extracellular matrix dysregulation, have also been described in AE transcriptomic data sets,^12^, ^13^, ^27^ including those from FLG-null and haplosufficient patients with AE,^12^, ^13^ reinforcing the utility of our disease model and underscoring the central role of FLG deficiency in AE pathophysiology.

Carefully controlled protease activity is essential for the maintenance of epidermal homeostasis and barrier function.^28^ In addition to increased expression of the chymotrypsin-like serine protease KLK7, expression of the cysteine protease CTSV and zinc-dependent protease CPA4 was significantly upregulated after FLG knockdown in the LSE model. KLK7 is highly expressed in skin,^29^ and its dysregulation has been implicated to AE pathogenesis; protein expression is increased in both involved and uninvolved AE skin and in the epidermis of flaky tail mice.^29^, ^30^, ^31^ Furthermore, overexpression of human KLK7 in murine models causes severe pruritus, increased epidermal thickness, and dermal inflammation.^30^ Our findings in AE skin are consistent with these results, demonstrating both increased overall epidermal KLK7 expression and ectopic expression within the lower epidermal compartments compared with those seen in healthy control skin. Importantly our novel *in vitro* data demonstrating increased *KLK7* mRNA and protein expression after FLG knockdown identifies for the first time that KLK7 is transcriptionally regulated, either directly or indirectly downstream of FLG (Figs 3 and 5). The combination of increased KLK7 expression and an atopic epidermal environment known to be permissive for protease activation^32^ has significant implications for barrier homeostasis. In addition to the perturbations in epidermal structure, enhanced KLK activity also drives inflammation both directly through IL-1 activation^33^ and indirectly through overexpression of thymic stromal lymphopoietin, a potent inducer and propagator of T_H_2-polarixed inflammation downstream of protease activated receptor 2 signaling.^34^ The mRNA expression of thymic stromal lymphopoietin is increased in lesional AE skin,^34^ flaky tail mice,^31^ and our LSEs after FLG knockdown (data not shown). This, combined with our observation that proteins of known immunomodulatory function (PPIA, TXN, ANXA3, and LY6D) are significantly perturbed by FLG knockdown, supports the idea that FLG deficiency can contribute to AE inflammation independently of immune cells.

PPIA is an ubiquitously expressed immunophilin family member protein with roles in protein folding, trafficking, and immune modulation.^35^ For the first time, we show that PPIA is downregulated in the context of FLG deficiency and in AE skin (Figs 3, 5, and 6), suggesting that PPIA might represent a novel disease mechanism downstream of FLG. Interestingly, PPIA is the cytoplasmic binding partner of cyclosporine, an effective therapeutic in patients with AE.^36^ Cyclosporine regulates gene transcription, growth, and differentiation of human keratinocytes,^37^ as well as their production of inflammatory cytokines.^38^ PPIA knockout mice experience spontaneous T_H_2-polarized allergic disease and an immune profile akin to AE, although no dermatitis is reported.^39^ No concurrent reduction in *PPIA* mRNA expression was observed in shFLG LSEs (Fig 5, *C*); the reduction in intracellular protein expression might instead reflect an increase in PPIA secretion known to be triggered after inflammatory/stress stimuli in epithelia.^35^ Extracellular PPIA functions as both a proinflammatory stimuli (autocrine/paracrine) and potent chemoattractant of CD147^+^ immune cells.^35^, ^40^ Stress and inflammatory pathways were overrepresented in our data set after FLG knockdown (Fig 4), and keratinocytes are known to secrete the close family member cyclophilin B and to express CD147 cell-surface receptors,^41^ suggesting that similar regulation of PPIA might occur within the epidermis. Furthermore, extracellular PPIA and TXN (increased in shFLG LSEs) have been identified as IgE-reactive self-antigens in patients with AE,^42^, ^43^ and therefore their dysregulation might represent an additional pathogenic mechanism downstream of FLG.

Finally, cyclophilin members are regulated during epithelial terminal differentiation,^44^ suggesting that PPIA dysregulation downstream of FLG might also have implications for epidermal barrier structure and function. Keratinocyte differentiation is associated with extensive keratin and actin remodeling.^45^ We also identified significantly differentially expressed proteins with known roles in cytoskeletal/actin remodeling (CFL1, CCT6A, and VIM) as novel targets of FLG loss. CFL1 is an essential actin-binding protein that regulates cell motility, growth, division, and differentiation by controlling actin filament assembly and disassembly.^46^ Overexpression of active (unphosphorylated) CFL1 in LSE models reduces corneocyte compaction, whereas partial knockdown increased proliferation, supporting a role for CFL1 in keratinocyte homeostasis.^47^ In our LSE model CFL1 protein but not mRNA expression was significantly increased after FLG knockdown (Fig 5). Pathways associated with regulation of the actin cytoskeleton have been reported to be transcriptionally overrepresented in AE skin,^13^ and *CFL1* mRNA expression is significantly upregulated in the involved skin of patients with AE, psoriasis, and lichen planus,^48^ suggesting that dysregulation of the actin cytoskeleton might be a contributing pathogenic mechanism. Interestingly, unlike PPIA and KLK7, the protein expression pattern of CFL1 in AE skin did not align to the shFLG LSE model, and we observed no increase in CFL1 protein expression in the patients with AE (Fig E6). The regulation of CFL1 is complex, involving phosphorylation, inhibitor complexes, and cooperation with other actin-binding proteins^46^; understanding its role in patients with AE will require further investigation.

Further IPA analysis significantly associated dysregulation of eIF2 (RPL22, RPL4, RPS27, and RPS5), eIF4 (RPS27 and RPS5), and mTOR (RPS27 and RPS5) signaling with FLG knockdown. Collectively, these pathways cooperate to regulate transcriptional and translational initiation in response to a wide variety of environmental stress stimuli, including infection.^49^, ^50^ Patients with AE have an increased susceptibility to both disseminated viral (eczema herpeticum) and bacterial skin infections,^51^ and the risk of both is increased in FLG-deficient subjects.^52^ Consistent with this, stress responses, namely type 1 interferon–mediated anti-pathogenic deference, are overrepresented in the transcriptome of FLG-null and haplosufficient patients with AE,^12^ and furthermore, type 1 interferon expression is increased in patients with eczema herpeticum.^53^ Type 1 interferons are secreted after viral^54^ or bacterial^55^ infection and mediate both immunomodulatory responses and local cell-cycle regulation in part through activation of eIF2,^56^ mTOR, and downstream p70-S6K/eIF4 signaling.^57^ For the first time, our *in vitro* data suggest that the exaggerated but ineffective upregulation of type 1 interferon–mediated defense observed in patients with AE^12^ might reflect an inherent defect downstream of FLG rather than/in addition to a deregulated response to pathogen exposure.^12^, ^53^ Therefore dysregulation of these pathways as a result of inherited or acquired FLG deficiency might contribute to the propensity of patients with AE to have bacterial and viral skin infections.

Overall, our data reinforce that loss of FLG is itself an important pathogenic factor in patients with AE, driving the development and exacerbation of barrier and immune dysfunction. Importantly, we have shown that loss of FLG alone in the absence of any systemic immune signals can disrupt epidermal homeostasis resulting in proteomic changes relevant to AE. Our data are consistent with the so-called “outside-to-inside” disease hypothesis and places FLG deficiency firmly upstream of protease, innate inflammatory, and cytoskeletal pathway dysregulation. The observed protein changes in AE skin occurred to a similar degree in FLG-depleted, *FLG* wild-type, and single- and double-allele mutated subjects, suggesting that the mechanism of FLG reduction (whether through genotype, cytokine effects, or a combination thereof) might not be important.

For the first time, we have identified 17 proteins that are significantly differentially expressed in the absence of FLG, including KLK7, PPIA, and CFL1. Confirmation of these results in independent LSE cultures validates the proteomics methodologies, interpretation and the biological reproducibility of our identified targets. Replication of PPIA and KLK7 expression profiles in skin biopsy specimens from patients with AE further underscores the utility of our disease model. To the best of our knowledge, this is the first study to implicate loss of PPIA resulting from FLG deficiency to AE pathogenesis and also to show that the known increase in KLK7 expression observed in AE skin can occur as a consequence of FLG loss. Finally, our pathway analysis suggests a possible role of FLG deficiency in regulating cell stress, including mTOR, eIF4, and eIF2 signaling, which is broadly consistent with RNA sequencing analysis of uninvolved FLG-deficient AE skin.^12^ Lack of correlation between the protein and mRNA expression profiles of CFL1 and PPIA emphasize the value of proteomic studies. Complementary transcriptomic studies will provide further understanding of the signaling mechanisms upstream of our observations. To this end, development of robust bioinformatic platforms for the integration of transcriptomic and proteomic data sets will be hugely valuable.

**Key messages**

• Loss of FLG in the absence of inflammation is sufficient to induce molecular changes characteristic of AE, including inflammatory, proteolytic, and cytoskeletal dysregulation.
• The immunophilin PPIA is a novel protein decreased in FLG-deficient LSEs and clinically active AE skin.
• The characteristic upregulation of KLK7 expression in patients with AE occurs downstream of FLG loss.
• Disconnect occurs between the epidermal proteome and transcriptome, highlighting the importance of proteomic studies for the identification of disease mechanisms.

## Supplementary data

Online Repository text

Tables E1-E7

Legends for Figures E1-E6
