# Supplementary material for: Proteomic analysis of filaggrin deficiency identifies molecular signatures characteristic of atopic eczema
Source: J Allergy Clin Immunol. 2017 Nov;140(5):1299–309. doi: 10.1016/j.jaci.2017.01.039 (PMC5667587; doi:10.1016/j.jaci.2017.01.039)
Supplement: Online Repository text [file mmc1.docx]

**Supplementary Methods:**

**Lentivirus (shRNA) production**

The HEK293T packaging cell line was used to produce replication incompetent, shRNA expressing, lentivirus. Briefly, cells were cultured in DMEM supplemented with 10% foetal calf serum. GIPZ lentiviral shRNAmir vectors were purchased from Open Biosystems (CO, USA) with a sequence targeting filaggrin (shFLG, V3LHS_369921), or a non-targeting control sequence (shNT, RHS4346, NS). HEK293T cells were co-transfected with pMD2.G envelope plasmid (5 μg), pCMVδ8.91 packaging plasmid (15 μg) and pGIPZ shRNA transfer plasmid (20 μg) using the calcium chloride precipitation method as previously described [1]. The following day media was replaced with 12 ml of enriched DMEM supplemented with 20% FCS and incubated for 2 further days before collecting the lentiviral particle containing supernatant. Lentivirus-containing media was cleared of cell debris by centrifugation (2000 rpm, 30 minutes, 4^o^C).

**Lentiviral Transduction of keratinocytes**

Passage 1 keratinocytes were transduced at 5—70% confluence in 6 well plates. Lentiviral aliquots were diluted 1:4 in naive DMEM, supplemented with hexadimethrine bromide (4 μg/ml) and added to wells. Plates were spin transduced at room temperature (600g, 1.5 hrs). Cells were washed and cultured in fresh complete Epilife media for 48 hours, to allow for the induction of transgene expression, before selection in puromycin supplemented media (1 μg/ml). Lentiviral work was carried out in accordance with safety requirements from the Health and Safety Executive.

**Living skin equivalent (LSE) culture**

LSEs were generated as described previously [1]. Briefly, 1 x 10^6^ NHEKs were seeded into 12mm diameter, 0.4 μM PCF Millicell ® cell culture inserts (Millipore) and cultured overnight in complete Epilife® medium adjusted to 1.5 mM calcium (high calcium Epilife^TM^) . LSEs were subsequently raised to the air-liquid interface (ALI) and outer media was replaced with fresh high calcium Epilife® supplemented with 5 μg/ml ascorbic acid. Outer medium was changed on alternate days for a total of 14 days post ALI culture. LSEs were harvested on ice in pre-chilled PBS before removal from plastic insert and preparation for subsequent analysis.

**LSE protein extraction**

LSEs were resuspended in 150 μl of 8M Urea extraction buffer (8 M Urea, 50 mM Tris-HCL pH 8.0, 1 mM NaF, 1 mM Na_3_VO_4_, 1 mM PMSF, 0.25% SDS and 1x protease inhibitor cocktail (Sigma Aldrich, UK). The detached polycarbonate insert was removed and the LSE then homogenised on ice by sonication (3 x 10 seconds, 8 Hz). Insoluble material was removed by centrifugation (23,000 g, 20 minutes, 4^o^C).

**Immunoblotting and densitometry**

Protein concentration of urea lysates was determination by BCA assay (Pierce, Thermo-Fischer, UK). Protein lysates (20 μg) were resolved under reducing conditions using NOVEX 4-12% Bis-Tris gel system (Life Technologies, UK) and immunoblotted using standard protocols. Primary antibody conditions were: filaggrin clone 15C10, 1:200 (NCL-FLG, Novocastra)(antibody raised against the filaggrin repeat domain), cofilin-1, 1:500 (PA5-27627, Thermo Scientific), kallikrein-7, 1:1000 (AF2624, R&D systems), cyclophillin A, 1:300 (39-1100, Invitrogen), GAPDH, 1:10,000 (#2118, Cell Signaling Technology. Appropriate peroxidase conjugated secondary antibodies (Vectorlabs, UK) were diluted 1:5000. Immunoblots were developed chemoluminescently using film. Densitometry was performed using ImageJ and normalised to GAPDH.

**Immunohistochemistry**

Paraffin embedded LSE sections (4 μM) were stained with haemotoxylin and eosin (H&E) staining as per standard protocols [1]. Images were acquired on a Zeiss Axio-imager 2 microscope (Carl Zeiss Ltd, UK) and processed using Adobe Photoshop CS3 (Adobe Corp.).

**Immunofluorescence**

OCT-embedded LSE cryo-sections (4 μM) were fixed and stained as described in [2]. Primary antibody conditions were: filaggrin (Acetone fix, 1:100, MS-449-P1, Lab Vision-Neomarkers), cofilin-1 (paraformaldehyde fix, 1:300, PA5-27627, Thermo Scientific), kallikrein-7 (Methanol fix, 1:250, AF2624, R&D systems), cyclophillin A (paraformaldehyde fix, 1:500, 39-1100, Invitrogen). Appropriate fluorescently conjugated secondary antibodies (Alexa Fluor goat 568) were used in combination with Hoechst 33342 nuclear counterstain (Life Technologies). Slides were imaged under constant conditions using a Nikon A1 confocal microscope (x20 plus an additional 1.54x zoom) (Nikon Instruments Europe B.V, Netherlands). Images were processed using Photoshop CS5 software (Adobe Corp.).

Quantification was performed in image J; the total fluorescence density and area of manually defined regions of interest were calculated. Fluorescence intensity was defined as the fluorescence density per unit area. Background fluorescence was determined from the appropriate IgG control and negated from each calculation.

**Filaggrin sequencing**

Blood derived total DNA was analysed using Access Array 48.48 IFC (Integrated Fluidic Circuit) technology. 50ng of each DNA sample was enriched and amplified via PCR in the Access Array chip and subject to 48 validated, overlapping *FLG* specific Primer pair assays (500bp amplicons). This provided comprehensive coverage of the FLG coding regions including the extra filaggrin allelic repeats in at position 8 and 10. PCR products were harvested from the IFC array and quantified using an Agilent DNA 1000 Kit. After determining correct range of products was obtained, PCR products were pooled and purified using Agencourt® AMPure® XP Reagent Beads (Beckman Coulter Genomics). This pool was sequenced on Illumina MiSeq platform (2x250bp read mode). The raw sequence files were processed by a custom pipeline implemented in Pipeline Pilot ([www.accelrys.com](http://www.accelrys.com)) and input reads were mapped to a filaggrin in house reference sequence containing 12 repeats, introns, 5’and 3’ UTRs. The Mapper (version 0.7.10--‐r789) with the following parameters: gap opening penalty = 8, gap extension, Penalty = 2, mismatch penalty = 4, match score = 1 and minimum score threshold= 100. The Mapped reads are stored as indexed bam files which are then processed with the GATK toolkit (version v3.4-46-gbc02625, Java Version 1.7.0_75). The HaplotypeCaller module was used to calculate SNPs and indels for each sample separately using the following settings: minimum variant quality = 10, minimum pass quality = 50, maximum deletion length =12 and coverage was down sampled to 500 in high coverage regions. The individual gVCF files for each sample were then collectively processed with the Genotype GVCFs module to produce a joint VCF report for multiple sample (same quality cut offs). This approach is Flexible and scalable and increases the sensitivity of SNP detection. FLG mutational analysis was performed by the Institute of Medical Biology Group (A*STAR Singapore) by contractual agreement. A summary of the sequencing results is provided in Table E1.

**Proteomic Analysis**

**In-gel digest**

The whole lane was excised and spilt into 22 bands/ 22 samples cut in small pieces (1x1mm cubes) and the standard reduction, alkylation and trypsin (Promega) digestion protocol was performed as per this Nature methods publication [3].

**In-solution digest**

The lysates were reduced, alkylated and digested as per [4]. After digestion, the samples were desalted using StageTips before MS analysis [5].

**MS analysis**

Two different systems were used in the analysis of these samples. The in-gel digested samples were analysed on a LTQ-Orbitrap XL (Thermo Fisher Scientific) via a NanoFlex electrospray ion source (Proxeon) coupled to an easy-nLC 1000 (Thermo Fisher Scientific) using an Acclaim PepMap 100 75umx2cm nanoViper pre-column (Thermo Fisher Scientific) and an Acclaim PepMap 75umx15cm nanoViper C18, 2um, 100A (Thermo Fisher Scientific) chromatography column. The in-solution samples were run on a QExactive (Thermo Fisher Scientific) via an EasySpray nanoelectrospray ion source (Thermo Fisher Scientific) coupled to an easy-nLC 1000 (Thermo Fisher Scientific) using a Acclaim PepMap 100 75umx2cm nanoViper pre-column (Thermo Fisher Scientific) and an Easy PepMap 75umx50cm C18, 2um, 100A chromatography column (Thermo Fisher Scientific). In both cases the mobile phases were 0.1% Formic acid (Thermo Fisher Scientific) in water (Fluka) for the aqueous phase and Acetonitrile (Fluka) for the organic phase. The gradient time was 1hr for the Orbitrap XL run and the instrument was set to operate in data-dependent mode measuring both a full scan MS in the Orbitrap and the top 5 most intense ions in the LTQ after collisionally induced dissociation.

The Qexactive gradient time was 2.5hrs and column temperature was set to 40^o^C, the MS data was acquired using a Top 10 method using HCD fragmentation to sequence the top 10 ions per full scan spectrum and the instrument was set to 2% underfill ratio, and 3m/z isolation window [6].

**Data processing and Analysis**

Data analysis was performed with Thermo Proteome discoverer version 1.3 using Mascot (Matrix Science, London, UK) and the uniprot human database and its decoy, the false discovery rate was set to 1% and a label free quantification node, the other parameters were as per [7].

The samples were then further processed using Scaffold (Proteome Software Inc., Portland, OR) version 3.6.1 where proteome discoverer files were imported and reprocessed using X! Tandem (The GPM, thegpm.org; version CYCLONE (2010.12.01.1)). Protein identifications were accepted if they could be established at greater than 99.9% probability and contained at least 2 identified peptides. Protein probabilities were assigned by the Protein Prophet [8].

Proteomic analysis of the technical replicates identified 1,640 unique proteins. Given the high number of missing values the dataset was filtered to include only those proteins reported in 15 or more of the 20 sample preparations. Filtering reduced the data suitable for statistical analysis to a subset of 367 unique proteins. Given the classic paired study design (i.e. 10 paired donor samples) no additional data normalisation was implemented. All protein expression values were log_10_ transformed and the difference between the shFLG and shNT un-weighted spectrum counts for each protein were calculated for each of the 367 proteins. A one sample t-Test was applied to each of the estimated differences, with the null hypothesis that the mean of the differences was zero. Log ratios with an adjusted p-value of ˂0.05 were considered significantly differentially expressed and used as input for Ingenuity pathway analysis (IPA) and further downstream validation. All statistical analysis was completed using R version 3.1.2.

**Ingenuity Pathway Analysis**

Ingenuity pathway analysis (IPA) software (Qiagen, Redwood city, USA) was employed to assist with the biological interpretation of the significantly altered data set. Differentially regulated proteins were grouped by cellular function, disease relevance and canonical pathway involvement based on an extensive hand curated IPA knowledge base of human and rodent studies. A right-tailed Fisher’s exact test was implemented by IPA to determine if the representation of our overlaid proteins within each group/pathway was considered significant (P≤0.05). This takes into account both the number of imputed focus proteins that participate within each group/pathway and the respective total number of proteins known to be associated within the reference set (<http://www.ingenuity.com>).

**Reverse transcription-qPCR**

RNA was extracted from LSEs using the ReliaPrep miniprep system following manufacturer’s instructions (Promega). cDNA was prepared from 0.5 - 2 μg of total RNA using random priming. Moloney murine leukemia virus enzyme (MMLV) (100 units) and buffer system was used in a 20 µl reaction volume according to manufacturer’s instructions with the addition of RNAse OUT (5 units) (Life technologies).
The following exon-spanning probe based assays were used: filaggrin (Hs.PT.53a.23095403, FAM/TAMRA, Integrated DNA technologies), cyclophilin A (F: ATGCTGGACCCAACACAAAT, R: TCTTTCACTTTGCCAAACACC, UPL PROBE#48 FAM/NFQ, Roche), cofilin-1 (F: GTGCCCTCTCCTTTTCGTTT, R: TTGAACACCTTGATGACACCAT, UPL PROBE#5 FAM/NFQ, Roche), kallikrein-7 (Hs.PT.58.39819237, FAM/TAMRA, IDT, IA, USA). 18S was used as a housekeeping gene for normalisation purposes [9], in combination with Taqman gene expression mastermix according to manufacturer’s instructions (Life Technologies, UK). Assays were run on a StepOne plus qPCR machine (Life Technologies) and fold changes derived via the 2(-Delta Delta C(T)) method.

**Statistical analysis**

Statistical analysis was performed using prism 5 (GraphPad software, San Diego, CA). For paired comparisons between shNT and shFLG LSEs, data was expressed as a log_10_ fold change relative to shNT and a paired one sample T.Test was performed. For comparisons between normal, AE uninvolved and AE involved skin, data was expressed as a log_10_ fold change relative to normal and a one way Anova with Tukey post test was performed.

1. Forrester, A.R., M.S. Elias, E.L. Woodward, M. Graham, F.M. Williams, and N.J. Reynolds, *Induction of a chloracne phenotype in an epidermal equivalent model by 2,3,7,8-tetrachlorodibenzo-p-dioxin (TCDD) is dependent on aryl hydrocarbon receptor activation and is not reproduced by aryl hydrocarbon receptor knock down.* J Dermatol Sci, 2014. **73**(1): p. 10-22.

2. Flockhart, R.J., B.L. Diffey, P.M. Farr, J. Lloyd, and N.J. Reynolds, *NFAT regulates induction of COX-2 and apoptosis of keratinocytes in response to ultraviolet radiation exposure.* FASEB J, 2008. **22**(12): p. 4218-27.

3. Shevchenko, A., H. Tomas, J. Havlis, J.V. Olsen, and M. Mann, *In-gel digestion for mass spectrometric characterization of proteins and proteomes.* Nat Protoc, 2006. **1**(6): p. 2856-60.

4. Lopez-Ferrer, D., A. Ramos-Fernandez, S. Martinez-Bartolome, P. Garcia-Ruiz, and J. Vazquez, *Quantitative proteomics using 16O/18O labeling and linear ion trap mass spectrometry.* Proteomics, 2006. **6 Suppl 1**: p. S4-11.

5. Rappsilber, J., Y. Ishihama, and M. Mann, *Stop and go extraction tips for matrix-assisted laser desorption/ionization, nanoelectrospray, and LC/MS sample pretreatment in proteomics.* Anal Chem, 2003. **75**(3): p. 663-70.

6. Scheltema, R.A., J.P. Hauschild, O. Lange, D. Hornburg, E. Denisov, E. Damoc, et al., *The Q Exactive HF, a Benchtop mass spectrometer with a pre-filter, high-performance quadrupole and an ultra-high-field Orbitrap analyzer.* Mol Cell Proteomics, 2014. **13**(12): p. 3698-708.

7. Azimzadeh, O., H. Scherthan, H. Sarioglu, Z. Barjaktarovic, M. Conrad, A. Vogt, et al., *Rapid proteomic remodeling of cardiac tissue caused by total body ionizing radiation.* Proteomics, 2011. **11**(16): p. 3299-311.

8. Nesvizhskii, A.I., A. Keller, E. Kolker, and R. Aebersold, *A statistical model for identifying proteins by tandem mass spectrometry.* Anal Chem, 2003. **75**(17): p. 4646-58.

9. Litherland, G.J., C. Dixon, R.L. Lakey, T. Robson, D. Jones, D.A. Young, et al., *Synergistic collagenase expression and cartilage collagenolysis are phosphatidylinositol 3-kinase/Akt signaling-dependent.* J Biol Chem, 2008. **283**(21): p. 14221-9.
