# Supplementary material for: Proteomic analysis of filaggrin deficiency identifies molecular signatures characteristic of atopic eczema
Source: J Allergy Clin Immunol. 2017 Nov;140(5):1299–309. doi: 10.1016/j.jaci.2017.01.039 (PMC5667587; doi:10.1016/j.jaci.2017.01.039)
Supplement: Tables E1-E7 [file mmc2.docx]

**Table E1: List of 360 proteins common to ≥ 75% of analysed samples**

| Uniprot ID | | Protein name | | | | | | Mean difference | | | | | | P-value | | | | | | | |  |  |  |  |
| --- | --- | --- | --- | --- | --- | --- | --- | --- | --- | --- | --- | --- | --- | --- | --- | --- | --- | --- | --- | --- | --- | --- | --- | --- | --- |
| A2ML1_HUMAN | | alpha-2-macroglobulin-like 1 | | | | | | -0.02 | | | | | | 0.73 | | | | | | | |  |  |  |  |
| ACADV_HUMAN | | acyl-CoA dehydrogenase, very long chain | | | | | | -0.15 | | | | | | 0.11 | | | | | | | |  |  |  |  |
| THIC_HUMAN | | acetyl-CoA acetyltransferase 2 | | | | | | -0.08 | | | | | | 0.48 | | | | | | | |  |  |  |  |
| ACLY_HUMAN | | ATP citrate lyase | | | | | | -0.08 | | | | | | 0.55 | | | | | | | |  |  |  |  |
| ACON_HUMAN | | aconitase 2, mitochondrial | | | | | | -0.03 | | | | | | 0.79 | | | | | | | |  |  |  |  |
| ACTBL_HUMAN | | actin, beta-like 2 | | | | | | -0.06 | | | | | | 0.25 | | | | | | | |  |  |  |  |
| ACTN4_HUMAN | | actinin, alpha 4 | | | | | | -0.07 | | | | | | 0.21 | | | | | | | |  |  |  |  |
| ACTZ_HUMAN | | ARP1 actin-related protein 1 homolog A, centractin alpha (yeast) | | | | | | 0.11 | | | | | | 0.47 | | | | | | | |  |  |  |  |
| ARP3_HUMAN | | ARP3 actin-related protein 3 homolog (yeast) | | | | | | 0.05 | | | | | | 0.57 | | | | | | | |  |  |  |  |
| AHNK_HUMAN | | AHNAK nucleoprotein | | | | | | 0.12 | | | | | | 0.16 | | | | | | | |  |  |  |  |
| AHNK2_HUMAN | | AHNAK nucleoprotein 2 | | | | | | 0.08 | | | | | | 0.47 | | | | | | | |  |  |  |  |
| ALDH2_HUMAN | | aldehyde dehydrogenase 2 family (mitochondrial) | | | | | | 0.16 | | | | | | 0.14 | | | | | | | |  |  |  |  |
| ALDOA_HUMAN | | aldolase A, fructose-bisphosphate | | | | | | 0 | | | | | | 0.95 | | | | | | | |  |  |  |  |
| ALDOC_HUMAN | | aldolase C, fructose-bisphosphate | | | | | | 0.07 | | | | | | 0.58 | | | | | | | |  |  |  |  |
| ANXA1_HUMAN | | annexin A1 | | | | | | 0.07 | | | | | | 0.14 | | | | | | | |  |  |  |  |
| ANXA2_HUMAN | | annexin A2 | | | | | | 0.04 | | | | | | 0.25 | | | | | | | |  |  |  |  |
| ANXA3_HUMAN | | annexin A3 | | | | | | 0.17 | | | | | | 0.02 | | | | | | | |  |  |  |  |
| ANXA4_HUMAN | | annexin A4 | | | | | | -0.06 | | | | | | 0.44 | | | | | | | |  |  |  |  |
| ANXA5_HUMAN | | annexin A5 | | | | | | 0.02 | | | | | | 0.83 | | | | | | | |  |  |  |  |
| ANXA8_HUMAN | | annexin A8-like 1 | | | | | | -0.06 | | | | | | 0.56 | | | | | | | |  |  |  |  |
| AP1B1_HUMAN | | adaptor-related protein complex 1, beta 1 subunit | | | | | | 0.12 | | | | | | 0.28 | | | | | | | |  |  |  |  |
| ACPH_HUMAN | | acylaminoacyl-peptide hydrolase | | | | | | -0.2 | | | | | | 0.14 | | | | | | | |  |  |  |  |
| ARF1_HUMAN | | ADP-ribosylation factor 1 | | | | | | -0.05 | | | | | | 0.46 | | | | | | | |  |  |  |  |
| ARF4_HUMAN | | ADP-ribosylation factor 4 | | | | | | 0.11 | | | | | | 0.34 | | | | | | | |  |  |  |  |
| ARGI1_HUMAN | | arginase 1 | | | | | | 0.03 | | | | | | 0.59 | | | | | | | |  |  |  |  |
| ARPC4_HUMAN | | actin related protein 2/3 complex, subunit 4, 20kDa | | | | | | 0.03 | | | | | | 0.78 | | | | | | | |  |  |  |  |
| APRV1_HUMAN | | aspartic peptidase, retroviral-like 1 | | | | | | -0.05 | | | | | | 0.59 | | | | | | | |  |  |  |  |
| PUR9_HUMAN | | 5-aminoimidazole-4-carboxamide ribonucleotide formyltransferase/IMP cyclohydrolase | | | | | | -0.08 | | | | | | 0.56 | | | | | | | |  |  |  |  |
| AT1A1_HUMAN | | ATPase, Na+/K+ transporting, alpha 1 polypeptide | | | | | | 0.07 | | | | | | 0.43 | | | | | | | |  |  |  |  |
| AT2A2_HUMAN | ATPase, Ca++ transporting, cardiac muscle, slow twitch 2 | | | | | | -0.17 | | | | | | 0.17 | | | | | | | |  |  |  |  |  |
| ATPA_HUMAN | ATP synthase, H+ transporting, mitochondrial F1 complex, alpha subunit 1, cardiac muscle | | | | | | -0.01 | | | | | | 0.80 | | | | | | | |  |  |  |  |  |
| ATPB_HUMAN | ATP synthase, H+ transporting, mitochondrial F1 complex, beta polypeptide | | | | | | -0.01 | | | | | | 0.89 | | | | | | | |  |  |  |  |  |
| ATPO_HUMAN | ATP synthase, H+ transporting, mitochondrial F1 complex, O subunit | | | | | | -0.06 | | | | | | 0.32 | | | | | | | |  |  |  |  |  |
| VATA_HUMAN | ATPase, H+ transporting, lysosomal 70kDa, V1 subunit A | | | | | | 0.07 | | | | | | 0.42 | | | | | | | |  |  |  |  |  |
| VATB2_HUMAN | ATPase, H+ transporting, lysosomal 56/58kDa, V1 subunit B2 | | | | | | -0.08 | | | | | | 0.51 | | | | | | | |  |  |  |  |  |
| ZA2G_HUMAN | alpha-2-glycoprotein 1, zinc-binding | | | | | | 0.01 | | | | | | 0.92 | | | | | | | |  |  |  |  |  |
| BAP31_HUMAN | B-cell receptor-associated protein 31 | | | | | | 0.09 | | | | | | 0.42 | | | | | | | |  |  |  |  |  |
| BLMH_HUMAN | bleomycin hydrolase | | | | | | 0.02 | | | | | | 0.83 | | | | | | | |  |  |  |  |  |
| C1QBP_HUMAN | complement component 1, q subcomponent binding protein | | | | | | -0.06 | | | | | | 0.36 | | | | | | | |  |  |  |  |  |
| CAH2_HUMAN | carbonic anhydrase II | | | | | | -0.06 | | | | | | 0.59 | | | | | | | |  |  |  |  |  |
| CALL3_HUMAN | calmodulin-like 3 | | | | | | 0.08 | | | | | | 0.12 | | | | | | | |  |  |  |  |  |
| CALL5_HUMAN | calmodulin-like 5 | | | | | | 0.05 | | | | | | 0.41 | | | | | | | |  |  |  |  |  |
| CALR_HUMAN | calreticulin | | | | | | -0.02 | | | | | | 0.82 | | | | | | | |  |  |  |  |  |
| CAND1_HUMAN | cullin-associated and neddylation-dissociated 1 | | | | | | 0.13 | | | | | | 0.27 | | | | | | | |  |  |  |  |  |
| CALX_HUMAN | calnexin | | | | | | 0.08 | | | | | | 0.28 | | | | | | | |  |  |  |  |  |
| CAP1_HUMAN | CAP, adenylate cyclase-associated protein 1 (yeast) | | | | | | 0.1 | | | | | | 0.08 | | | | | | | |  |  |  |  |  |
| CAPG_HUMAN | capping protein (actin filament), gelsolin-like | | | | | | 0.11 | | | | | | 0.25 | | | | | | | |  |  |  |  |  |
| CAN1_HUMAN | calpain 1, (mu/I) large subunit | | | | | | -0.02 | | | | | | 0.85 | | | | | | | |  |  |  |  |  |
| CAZA1_HUMAN | capping protein (actin filament) muscle Z-line, alpha 1 | | | | | | 0.04 | | | | | | 0.70 | | | | | | | |  |  |  |  |  |
| CAPZB_HUMAN | capping protein (actin filament) muscle Z-line, beta | | | | | | 0.03 | | | | | | 0.74 | | | | | | | |  |  |  |  |  |
| CASPE_HUMAN | caspase 14, apoptosis-related cysteine peptidase | | | | | | 0.08 | | | | | | 0.14 | | | | | | | |  |  |  |  |  |
| ICAL_HUMAN | calpastatin | | | | | | 0.04 | | | | | | 0.75 | | | | | | | |  |  |  |  |  |
| CATA_HUMAN | catalase | | | | | | -0.01 | | | | | | 0.91 | | | | | | | |  |  |  |  |  |
| CBR1_HUMAN | carbonyl reductase 1 | | | | | | 0.02 | | | | | | 0.85 | | | | | | | |  |  |  |  |  |
| TCPB_HUMAN | chaperonin containing TCP1, subunit 2 (beta) | | | | | | 0.05 | | | | | | 0.34 | | | | | | | |  |  |  |  |  |
| TCPG_HUMAN | chaperonin containing TCP1, subunit 3 (gamma) | | | | | | 0.05 | | | | | | 0.65 | | | | | | | |  |  |  |  |  |
| TCPD_HUMAN | chaperonin containing TCP1, subunit 4 (delta) | | | | | | 0.02 | | | | | | 0.87 | | | | | | | |  |  |  |  |  |
| TCPE_HUMAN | chaperonin containing TCP1, subunit 5 (epsilon) | | | | | | -0.06 | | | | | | 0.44 | | | | | | | |  |  |  |  |  |
| TCPZ_HUMAN | | | | | chaperonin containing TCP1, subunit 6A (zeta 1) | | | | | | -0.11 | | | | | | 0.01 | | |  |  |  |  |  |  |
| TCPH_HUMAN | | | | | chaperonin containing TCP1, subunit 7 (eta) | | | | | | -0.07 | | | | | | 0.21 | | |  |  |  |  |  |  |
| TCPQ_HUMAN | | | | | chaperonin containing TCP1, subunit 8 (theta) | | | | | | -0.01 | | | | | | 0.94 | | |  |  |  |  |  |  |
| COF1_HUMAN | | | | | cofilin 1 (non-muscle) | | | | | | 0.11 | | | | | | 0.03 | | |  |  |  |  |  |  |
| CKAP4_HUMAN | | | | | cytoskeleton-associated protein 4 | | | | | | 0.06 | | | | | | 0.55 | | |  |  |  |  |  |  |
| CLIC1_HUMAN | | | | | chloride intracellular channel 1 | | | | | | 0.05 | | | | | | 0.59 | | |  |  |  |  |  |  |
| CLIC3_HUMAN | | | | | chloride intracellular channel 3 | | | | | | -0.02 | | | | | | 0.87 | | |  |  |  |  |  |  |
| CLH1_HUMAN | | | | | clathrin, heavy chain (Hc) | | | | | | -0.04 | | | | | | 0.60 | | |  |  |  |  |  |  |
| COHA1_HUMAN | | | | | collagen, type XVII, alpha 1 | | | | | | 0.13 | | | | | | 0.10 | | |  |  |  |  |  |  |
| COPB2_HUMAN | | | | | coatomer protein complex, subunit beta 2 (beta prime) | | | | | | 0.04 | | | | | | 0.78 | | |  |  |  |  |  |  |
| CBPA4_HUMAN | | | | | carboxypeptidase A4 | | | | | | 0.25 | | | | | | 0.04 | | |  |  |  |  |  |  |
| CISY_HUMAN | | | | | citrate synthase | | | | | | -0.17 | | | | | | 0.11 | | |  |  |  |  |  |  |
| CYTM_HUMAN | | | | | cystatin E/M | | | | | | 0.05 | | | | | | 0.65 | | |  |  |  |  |  |  |
| CYTA_HUMAN | | | | | cystatin A (stefin A) | | | | | | 0.03 | | | | | | 0.72 | | |  |  |  |  |  |  |
| CYTB_HUMAN | | | | | cystatin B (stefin B) | | | | | | 0.04 | | | | | | 0.80 | | |  |  |  |  |  |  |
| CTNA1_HUMAN | | | | | catenin (cadherin-associated protein), alpha 1, 102kDa | | | | | | 0.02 | | | | | | 0.83 | | |  |  |  |  |  |  |
| CATC_HUMAN | | | | | cathepsin C | | | | | | 0.04 | | | | | | 0.66 | | |  |  |  |  |  |  |
| CATD_HUMAN | | | | | cathepsin D | | | | | | 0 | | | | | | 0.96 | | |  |  |  |  |  |  |
| CATL2_HUMAN | | | | | cathepsin V | | | | | | 0.15 | | | | | | 0.05 | | |  |  |  |  |  |  |
| SRC8_HUMAN | | | | | cortactin | | | | | | 0.03 | | | | | | 0.55 | | |  |  |  |  |  |  |
| DDX3X_HUMAN | | | | | DEAD (Asp-Glu-Ala-Asp) box helicase 3, X-linked | | | | | | -0.04 | | | | | | 0.50 | | |  |  |  |  |  |  |
| DHCR7_HUMAN | | | | | 7-dehydrocholesterol reductase | | | | | | 0.07 | | | | | | 0.67 | | |  |  |  |  |  |  |
| DHX9_HUMAN | | | | | DEAH (Asp-Glu-Ala-His) box helicase 9 | | | | | | 0 | | | | | | 0.98 | | |  |  |  |  |  |  |
| DLDH_HUMAN | | | | | dihydrolipoamide dehydrogenase | | | | | | -0.01 | | | | | | 0.78 | | |  |  |  |  |  |  |
| DMKN_HUMAN | | | | | dermokine | | | | | | -0.07 | | | | | | 0.44 | | |  |  |  |  |  |  |
| DSC1_HUMAN | | | | | desmocollin 1 | | | | | | 0.03 | | | | | | 0.55 | | |  |  |  |  |  |  |
| DSC2_HUMAN | | | | | desmocollin 2 | | | | | | 0.11 | | | | | | 0.38 | | |  |  |  |  |  |  |
| DSC3_HUMAN | | | | | desmocollin 3 | | | | | | 0.07 | | | | | | 0.26 | | |  |  |  |  |  |  |
| DSG1_HUMAN | | | | | desmoglein 1 | | | | | | 0.05 | | | | | | 0.45 | | |  |  |  |  |  |  |
| DSG3_HUMAN | | | | | desmoglein 3 | | | | | | 0.03 | | | | | | 0.70 | | |  |  |  |  |  |  |
| DESP_HUMAN | desmoplakin | | | | | | -0.02 | | | | | | 0.69 | | | | | | | |  |  |  |  |  |
| DYHC1_HUMAN | dynein, cytoplasmic 1, heavy chain 1 | | | | | | -0.03 | | | | | | 0.85 | | | | | | | |  |  |  |  |  |
| ECH1_HUMAN | enoyl CoA hydratase 1, peroxisomal | | | | | | 0.1 | | | | | | 0.39 | | | | | | | |  |  |  |  |  |
| EF1A3_HUMAN | eukaryotic translation elongation factor 1 alpha 1 pseudogene 5 | | | | | | -0.12 | | | | | | 0.06 | | | | | | | |  |  |  |  |  |
| EF1B_HUMAN | eukaryotic translation elongation factor 1 beta 2 | | | | | | 0.06 | | | | | | 0.47 | | | | | | | |  |  |  |  |  |
| EF1D_HUMAN | eukaryotic translation elongation factor 1 delta (guanine nucleotide exchange protein) | | | | | | 0.01 | | | | | | 0.90 | | | | | | | |  |  |  |  |  |
| EF1G_HUMAN | eukaryotic translation elongation factor 1 gamma | | | | | | -0.07 | | | | | | 0.44 | | | | | | | |  |  |  |  |  |
| EF2_HUMAN | eukaryotic translation elongation factor 2 | | | | | | -0.03 | | | | | | 0.22 | | | | | | | |  |  |  |  |  |
| IF2G_HUMAN | eukaryotic translation initiation factor 2, subunit 3 gamma, 52kDa | | | | | | 0.04 | | | | | | 0.80 | | | | | | | |  |  |  |  |  |
| EIF3B_HUMAN | eukaryotic translation initiation factor 3, subunit B | | | | | | -0.03 | | | | | | 0.84 | | | | | | | |  |  |  |  |  |
| IF4A1_HUMAN | eukaryotic translation initiation factor 4A1 | | | | | | -0.07 | | | | | | 0.46 | | | | | | | |  |  |  |  |  |
| IF4G1_HUMAN | eukaryotic translation initiation factor 4 gamma, 1 | | | | | | -0.05 | | | | | | 0.69 | | | | | | | |  |  |  |  |  |
| IF6_HUMAN | eukaryotic translation initiation factor 6 | | | | | | 0.06 | | | | | | 0.49 | | | | | | | |  |  |  |  |  |
| ENOA_HUMAN | enolase 1, (alpha) | | | | | | -0.02 | | | | | | 0.74 | | | | | | | |  |  |  |  |  |
| EPIPL_HUMAN | epiplakin 1 | | | | | | -0.08 | | | | | | 0.24 | | | | | | | |  |  |  |  |  |
| SYEP_HUMAN | glutamyl-prolyl-tRNA synthetase | | | | | | -0.03 | | | | | | 0.66 | | | | | | | |  |  |  |  |  |
| ERP29_HUMAN | endoplasmic reticulum protein 29 | | | | | | -0.06 | | | | | | 0.55 | | | | | | | |  |  |  |  |  |
| EVPL_HUMAN | envoplakin | | | | | | 0.06 | | | | | | 0.51 | | | | | | | |  |  |  |  |  |
| EZRI_HUMAN | ezrin | | | | | | -0.01 | | | | | | 0.74 | | | | | | | |  |  |  |  |  |
| FABP5_HUMAN | fatty acid binding protein 5 (psoriasis-associated) | | | | | | 0.03 | | | | | | 0.59 | | | | | | | |  |  |  |  |  |
| FAS_HUMAN | fatty acid synthase | | | | | | 0.01 | | | | | | 0.82 | | | | | | | |  |  |  |  |  |
| FILA_HUMAN | filaggrin | | | | | | -0.46 | | | | | | 0.00 | | | | | | | |  |  |  |  |  |
| FILA2_HUMAN | filaggrin family member 2 | | | | | | -0.01 | | | | | | 0.92 | | | | | | | |  |  |  |  |  |
| FLNA_HUMAN | filamin A, alpha | | | | | | -0.01 | | | | | | 0.86 | | | | | | | |  |  |  |  |  |
| FLNB_HUMAN | filamin B, beta | | | | | | 0 | | | | | | 0.98 | | | | | | | |  |  |  |  |  |
| FSCN1_HUMAN | fascin actin-bundling protein 1 | | | | | | -0.05 | | | | | | 0.49 | | | | | | | |  |  |  |  |  |
| GANAB_HUMAN | glucosidase, alpha; neutral AB | | | | | | 0.11 | | | | | | 0.31 | | | | | | | |  |  |  |  |  |
| G3P_HUMAN | glyceraldehyde-3-phosphate dehydrogenase | | | | | | -0.05 | | | | | | 0.37 | | | | | | | |  |  |  |  |  |
| SYG_HUMAN | glycyl-tRNA synthetase | | | | | | 0.01 | | | | | | 0.86 | | | | | | | |  |  |  |  |  |
| GDIB_HUMAN | GDP dissociation inhibitor 2 | | | | | | -0.04 | | | | | | 0.76 | | | | | | | |  |  |  |  |  |
| GGCT_HUMAN | | gamma-glutamylcyclotransferase | | | | | | 0.07 | | | | | | 0.18 | | | | | | | |  |  |  |  |
| GLTP_HUMAN | | glycolipid transfer protein | | | | | | 0.06 | | | | | | 0.62 | | | | | | | |  |  |  |  |
| DHE3_HUMAN | | glutamate dehydrogenase 1 | | | | | | -0.06 | | | | | | 0.48 | | | | | | | |  |  |  |  |
| SAP3_HUMAN | | GM2 ganglioside activator | | | | | | 0.02 | | | | | | 0.75 | | | | | | | |  |  |  |  |
| GBLP_HUMAN | | guanine nucleotide binding protein (G protein), beta polypeptide 2-like 1 | | | | | | 0.1 | | | | | | 0.12 | | | | | | | |  |  |  |  |
| AATC_HUMAN | | glutamic-oxaloacetic transaminase 1, soluble | | | | | | -0.03 | | | | | | 0.76 | | | | | | | |  |  |  |  |
| AATM_HUMAN | | glutamic-oxaloacetic transaminase 2, mitochondrial | | | | | | 0.16 | | | | | | 0.13 | | | | | | | |  |  |  |  |
| G6PI_HUMAN | | glucose-6-phosphate isomerase | | | | | | -0.04 | | | | | | 0.27 | | | | | | | |  |  |  |  |
| GSDMA_HUMAN | | gasdermin A | | | | | | 0.02 | | | | | | 0.85 | | | | | | | |  |  |  |  |
| GELS_HUMAN | | gelsolin | | | | | | 0 | | | | | | 0.94 | | | | | | | |  |  |  |  |
| GSTP1_HUMAN | | glutathione S-transferase pi 1 | | | | | | -0.07 | | | | | | 0.20 | | | | | | | |  |  |  |  |
| H2AY_HUMAN | | H2A histone family, member Y | | | | | | 0.08 | | | | | | 0.35 | | | | | | | |  |  |  |  |
| H2BFS_HUMAN | | H2B histone family, member S (pseudogene) | | | | | | 0.03 | | | | | | 0.46 | | | | | | | |  |  |  |  |
| ECHA_HUMAN | | hydroxyacyl-CoA dehydrogenase/3-ketoacyl-CoA thiolase/enoyl-CoA hydratase (trifunctional protein), alpha subunit | | | | | | -0.01 | | | | | | 0.91 | | | | | | | |  |  |  |  |
| ECHB_HUMAN | | hydroxyacyl-CoA dehydrogenase/3-ketoacyl-CoA thiolase/enoyl-CoA hydratase (trifunctional protein), beta subunit | | | | | | 0 | | | | | | 0.98 | | | | | | | |  |  |  |  |
| HUTH_HUMAN | | histidine ammonia-lyase | | | | | | -0.02 | | | | | | 0.80 | | | | | | | |  |  |  |  |
| H13_HUMAN | | histone cluster 1, H1d | | | | | | 0.14 | | | | | | 0.38 | | | | | | | |  |  |  |  |
| HXK1_HUMAN | | hexokinase 1 | | | | | | 0.02 | | | | | | 0.73 | | | | | | | |  |  |  |  |
| HMOX1_HUMAN | | heme oxygenase (decycling) 1 | | | | | | -0.15 | | | | | | 0.56 | | | | | | | |  |  |  |  |
| ROA1_HUMAN | | heterogeneous nuclear ribonucleoprotein A1 | | | | | | -0.05 | | | | | | 0.36 | | | | | | | |  |  |  |  |
| ROA2_HUMAN | | heterogeneous nuclear ribonucleoprotein A2/B1 | | | | | | 0.09 | | | | | | 0.44 | | | | | | | |  |  |  |  |
| HNRPC_HUMAN | | heterogeneous nuclear ribonucleoprotein C (C1/C2) | | | | | | 0.06 | | | | | | 0.26 | | | | | | | |  |  |  |  |
| HNRH1_HUMAN | | heterogeneous nuclear ribonucleoprotein H1 (H) | | | | | | 0 | | | | | | 0.99 | | | | | | | |  |  |  |  |
| HNRPK_HUMAN | | heterogeneous nuclear ribonucleoprotein K | | | | | | -0.05 | | | | | | 0.48 | | | | | | | |  |  |  |  |
| HNRPM_HUMAN | | heterogeneous nuclear ribonucleoprotein M | | | | | | 0.12 | | | | | | 0.24 | | | | | | | |  |  |  |  |
| HNRPU_HUMAN | | heterogeneous nuclear ribonucleoprotein U (scaffold attachment factor A) | | | | | | 0.06 | | | | | | 0.53 | | | | | | | |  |  |  |  |
| HCD2_HUMAN | | hydroxysteroid (17-beta) dehydrogenase 10 | | | | | | -0.24 | | | | | | 0.07 | | | | | | | |  |  |  |  |
| DHB4_HUMAN | | hydroxysteroid (17-beta) dehydrogenase 4 | | | | | | -0.03 | | | | | | 0.73 | | | | | | | |  |  |  |  |
| ENPL_HUMAN | | heat shock protein 90kDa beta (Grp94), member 1 | | | | | | 0.05 | | | | | | 0.27 | | | | | | | |  |  |  |  |
| HSP74_HUMAN | | heat shock 70kDa protein 4 | | | | | | 0.01 | | | | | | 0.96 | | | | | | | |  |  |  |  |
| HS74L_HUMAN | | | heat shock 70kDa protein 4-like | | | | | | -0.01 | | | | | | 0.97 | | | | | | | |  |  |  |
| HSP7C_HUMAN | | | heat shock 70kDa protein 8 | | | | | | 0.03 | | | | | | 0.51 | | | | | | | |  |  |  |
| GRP75_HUMAN | | | heat shock 70kDa protein 9 (mortalin) | | | | | | 0.1 | | | | | | 0.16 | | | | | | | |  |  |  |
| HSPB1_HUMAN | | | heat shock 27kDa protein 1 | | | | | | -0.11 | | | | | | 0.05 | | | | | | | |  |  |  |
| CH60_HUMAN | | | heat shock 60kDa protein 1 (chaperonin) | | | | | | 0.02 | | | | | | 0.71 | | | | | | | |  |  |  |
| CH10_HUMAN | | | heat shock 10kDa protein 1 | | | | | | -0.1 | | | | | | 0.22 | | | | | | | |  |  |  |
| HS105_HUMAN | | | heat shock 105kDa/110kDa protein 1 | | | | | | -0.08 | | | | | | 0.46 | | | | | | | |  |  |  |
| HTRA1_HUMAN | | | HtrA serine peptidase 1 | | | | | | 0.05 | | | | | | 0.66 | | | | | | | |  |  |  |
| HYOU1_HUMAN | | | hypoxia up-regulated 1 | | | | | | -0.05 | | | | | | 0.67 | | | | | | | |  |  |  |
| IDE_HUMAN | | | insulin-degrading enzyme | | | | | | -0.15 | | | | | | 0.06 | | | | | | | |  |  |  |
| IDHC_HUMAN | | | isocitrate dehydrogenase 1 (NADP+), soluble | | | | | | 0.01 | | | | | | 0.94 | | | | | | | |  |  |  |
| IL36G_HUMAN | | | interleukin 36, gamma | | | | | | -0.05 | | | | | | 0.71 | | | | | | | |  |  |  |
| ILF2_HUMAN | | | interleukin enhancer binding factor 2 | | | | | | -0.08 | | | | | | 0.28 | | | | | | | |  |  |  |
| IMDH2_HUMAN | | | IMP (inosine 5'-monophosphate) dehydrogenase 2 | | | | | | 0.09 | | | | | | 0.49 | | | | | | | |  |  |  |
| IQGA1_HUMAN | | | IQ motif containing GTPase activating protein 1 | | | | | | -0.01 | | | | | | 0.93 | | | | | | | |  |  |  |
| ITA6_HUMAN | | | integrin, alpha 6 | | | | | | 0.12 | | | | | | 0.24 | | | | | | | |  |  |  |
| ITB1_HUMAN | | | integrin, beta 1 (fibronectin receptor, beta polypeptide, antigen CD29 includes MDF2, MSK12) | | | | | | -0.22 | | | | | | 0.11 | | | | | | | |  |  |  |
| ITB4_HUMAN | | | integrin, beta 4 | | | | | | 0.02 | | | | | | 0.81 | | | | | | | |  |  |  |
| INVO_HUMAN | | | involucrin | | | | | | 0.02 | | | | | | 0.55 | | | | | | | |  |  |  |
| PLAK_HUMAN | | | junction plakoglobin | | | | | | -0.06 | | | | | | 0.27 | | | | | | | |  |  |  |
| KINH_HUMAN | | | kinesin family member 5B | | | | | | 0 | | | | | | 0.99 | | | | | | | |  |  |  |
| KLK10_HUMAN | | | kallikrein-related peptidase 10 | | | | | | 0.06 | | | | | | 0.61 | | | | | | | |  |  |  |
| KLK7_HUMAN | | | kallikrein-related peptidase 7 | | | | | | 0.34 | | | | | | 0.02 | | | | | | | |  |  |  |
| IMB1_HUMAN | | | karyopherin (importin) beta 1 | | | | | | -0.01 | | | | | | 0.91 | | | | | | | |  |  |  |
| KPRP_HUMAN | | | keratinocyte proline-rich protein | | | | | | 0.24 | | | | | | 0.13 | | | | | | | |  |  |  |
| K1C10_HUMAN | | | keratin 10 | | | | | | -0.07 | | | | | | 0.10 | | | | | | | |  |  |  |
| K22E_HUMAN | | | keratin 2 | | | | | | -0.07 | | | | | | 0.10 | | | | | | | |  |  |  |
| K2C80_HUMAN | | | keratin 80 | | | | | | -0.07 | | | | | | 0.54 | | | | | | | |  |  |  |
| K1C9_HUMAN | | | keratin 9 | | | | | | -0.07 | | | | | | 0.58 | | | | | | | |  |  |  |
| LAD1_HUMAN | | | ladinin 1 | | | | | | -0.02 | | | | | | 0.73 | | | | | | | |  |  |  |
| LAMP1_HUMAN | | | | | | lysosomal-associated membrane protein 1 | | | | | | 0.1 | | | | | | | 0.39 | | | | | | |
| NGAL_HUMAN | | | | | | lipocalin 2 | | | | | | -0.02 | | | | | | | 0.80 | | | | | | |
| LDHA_HUMAN | | | | | | lactate dehydrogenase A | | | | | | 0.03 | | | | | | | 0.60 | | | | | | |
| LDHB_HUMAN | | | | | | lactate dehydrogenase B | | | | | | 0.04 | | | | | | | 0.55 | | | | | | |
| LG3BP_HUMAN | | | | | | lectin, galactoside-binding, soluble, 3 binding protein | | | | | | 0.01 | | | | | | | 0.92 | | | | | | |
| LEG7_HUMAN | | | | | | lectin, galactoside-binding, soluble, 7 | | | | | | -0.11 | | | | | | | 0.17 | | | | | | |
| LMNA_HUMAN | | | | | | lamin A/C | | | | | | 0.05 | | | | | | | 0.33 | | | | | | |
| LMNB1_HUMAN | | | | | | lamin B1 | | | | | | -0.04 | | | | | | | 0.78 | | | | | | |
| LY6D_HUMAN | | | | | | lymphocyte antigen 6 complex, locus D | | | | | | 0.14 | | | | | | | 0.04 | | | | | | |
| MAP4_HUMAN | | | | | | microtubule-associated protein 4 | | | | | | 0.06 | | | | | | | 0.27 | | | | | | |
| MDHC_HUMAN | | | | | | malate dehydrogenase 1, NAD (soluble) | | | | | | 0.06 | | | | | | | 0.51 | | | | | | |
| MDHM_HUMAN | | | | | | malate dehydrogenase 2, NAD (mitochondrial) | | | | | | 0.02 | | | | | | | 0.65 | | | | | | |
| MAOX_HUMAN | | | | | | malic enzyme 1, NADP(+)-dependent, cytosolic | | | | | | 0.07 | | | | | | | 0.50 | | | | | | |
| MVP_HUMAN | | | | | | major vault protein | | | | | | 0.04 | | | | | | | 0.62 | | | | | | |
| MX1_HUMAN | | | | | | MX dynamin-like GTPase 1 | | | | | | -0.04 | | | | | | | 0.65 | | | | | | |
| MYH9_HUMAN | | | | | | myosin, heavy chain 9, non-muscle | | | | | | -0.06 | | | | | | | 0.16 | | | | | | |
| MYL6_HUMAN | | | | | | myosin, light chain 6, alkali, smooth muscle and non-muscle | | | | | | 0.04 | | | | | | | 0.61 | | | | | | |
| NUCL_HUMAN | | | | | | nucleolin | | | | | | 0.03 | | | | | | | 0.54 | | | | | | |
| NDKA_HUMAN | | | | | | NME/NM23 nucleoside diphosphate kinase 1 | | | | | | -0.15 | | | | | | | 0.27 | | | | | | |
| NPC2_HUMAN | | | | | | Niemann-Pick disease, type C2 | | | | | | 0.11 | | | | | | | 0.39 | | | | | | |
| PSA_HUMAN | | | | | | aminopeptidase puromycin sensitive | | | | | | 0.04 | | | | | | | 0.85 | | | | | | |
| NPM_HUMAN | | | | | | nucleophosmin (nucleolar phosphoprotein B23, numatrin) | | | | | | 0.07 | | | | | | | 0.18 | | | | | | |
| PDIA1_HUMAN | | | | | | prolyl 4-hydroxylase, beta polypeptide | | | | | | -0.02 | | | | | | | 0.58 | | | | | | |
| PA2G4_HUMAN | | | | | | proliferation-associated 2G4, 38kDa | | | | | | -0.03 | | | | | | | 0.64 | | | | | | |
| PABP1_HUMAN | | | | | | poly(A) binding protein, cytoplasmic 1 | | | | | | -0.02 | | | | | | | 0.85 | | | | | | |
| PARK7_HUMAN | | | | | | parkinson protein 7 | | | | | | 0.06 | | | | | | | 0.54 | | | | | | |
| PCBP1_HUMAN | | | | | | poly(rC) binding protein 1 | | | | | | 0.06 | | | | | | | 0.42 | | | | | | |
| PDC6I_HUMAN | | | | | | programmed cell death 6 interacting protein | | | | | | 0.07 | | | | | | | 0.33 | | | | | | |
| PDIA3_HUMAN | | | | | | protein disulfide isomerase family A, member 3 | | | | | | 0 | | | | | | | 0.96 | | | | | | |
| PDIA4_HUMAN | | | | | | protein disulfide isomerase family A, member 4 | | | | | | 0.02 | | | | | | | 0.77 | | | | | | |
| PDIA6_HUMAN | | | | | | protein disulfide isomerase family A, member 6 | | | | | | -0.03 | | | | | | | 0.73 | | | | | | |
| PDLI1_HUMAN | | | | | | PDZ and LIM domain 1 | | | | | | 0.09 | | | | | | | 0.53 | | | | | | |
| PEBP1_HUMAN | | | | | | phosphatidylethanolamine binding protein 1 | | | | | | 0.05 | | | | | | | 0.57 | | | | | | |
| PROF1_HUMAN | | | | | | profilin 1 | | | | | | 0 | | | | | | | 0.96 | | | | | | |
| PGAM1_HUMAN | | | | | | phosphoglycerate mutase 1 (brain) | | | | | | 0.03 | | | | | | | 0.61 | | | | | | |
| 6PGD_HUMAN | | | | | | phosphogluconate dehydrogenase | | | | | | -0.09 | | | | | | | 0.48 | | | | | | |
| PGK1_HUMAN | | | | | | phosphoglycerate kinase 1 | | | | | | -0.07 | | | | | | | 0.37 | | | | | | |
| PHB_HUMAN | | | | | | prohibitin | | | | | | 0.06 | | | | | | | 0.43 | | | | | | |
| PHB2_HUMAN | | | | | | prohibitin 2 | | | | | | -0.04 | | | | | | | 0.61 | | | | | | |
| SERA_HUMAN | | | | | | phosphoglycerate dehydrogenase | | | | | | 0 | | | | | | | 0.97 | | | | | | |
| ELAF_HUMAN | | | | | | peptidase inhibitor 3, skin-derived | | | | | | 0.1 | | | | | | | 0.26 | | | | | | |
| KPYM_HUMAN | | | | | | pyruvate kinase, muscle | | | | | | -0.02 | | | | | | | 0.63 | | | | | | |
| PKP1_HUMAN | | | | | | plakophilin 1 | | | | | | 0.02 | | | | | | | 0.72 | | | | | | |
| PKP3_HUMAN | | | | | | plakophilin 3 | | | | | | 0.04 | | | | | | | 0.48 | | | | | | |
| PLEC_HUMAN | | | | | | plectin | | | | | | -0.05 | | | | | | | 0.51 | | | | | | |
| PLST_HUMAN | | | | | | plastin 3 | | | | | | 0.02 | | | | | | | 0.61 | | | | | | |
| PNPH_HUMAN | | | | | | purine nucleoside phosphorylase | | | | | | 0.04 | | | | | | | 0.86 | | | | | | |
| POF1B_HUMAN | | | | | | premature ovarian failure, 1B | | | | | | -0.01 | | | | | | | 0.82 | | | | | | |
| PPIA_HUMAN | | | | | | peptidylprolyl isomerase A (cyclophilin A) | | | | | | -0.06 | | | | | | | 0.00 | | | | | | |
| PPIB_HUMAN | | | | | | peptidylprolyl isomerase B (cyclophilin B) | | | | | | 0.02 | | | | | | | 0.79 | | | | | | |
| PEPL_HUMAN | | | | | | periplakin | | | | | | 0.1 | | | | | | | 0.24 | | | | | | |
| 2AAA_HUMAN | | | | | | protein phosphatase 2, regulatory subunit A, alpha | | | | | | -0.06 | | | | | | | 0.46 | | | | | | |
| PRDX1_HUMAN | | | | | | peroxiredoxin 1 | | | | | | 0 | | | | | | | 0.98 | | | | | | |
| PRDX2_HUMAN | | | | | | peroxiredoxin 2 | | | | | | -0.01 | | | | | | | 0.89 | | | | | | |
| PRDX5_HUMAN | | | | | | peroxiredoxin 5 | | | | | | -0.04 | | | | | | | 0.78 | | | | | | |
| PRDX6_HUMAN | | | | | | peroxiredoxin 6 | | | | | | -0.04 | | | | | | | 0.69 | | | | | | |
| GLU2B_HUMAN | | | | | | protein kinase C substrate 80K-H | | | | | | 0.03 | | | | | | | 0.72 | | | | | | |
| PSA1_HUMAN | | | | | | proteasome (prosome, macropain) subunit, alpha type, 1 | | | | | | 0.1 | | | | | | | 0.35 | | | | | | |
| PSA2_HUMAN | | | | | | proteasome (prosome, macropain) subunit, alpha type, 2 | | | | | | 0.01 | | | | | | | 0.87 | | | | | | |
| PSA3_HUMAN | | | | | | proteasome (prosome, macropain) subunit, alpha type, 3 | | | | | | 0 | | | | | | | 0.99 | | | | | | |
| PSA4_HUMAN | | | | | proteasome (prosome, macropain) subunit, alpha type, 4 | | | | | | -0.06 | | | | | | | 0.52 | | | | | | |  |
| PSA5_HUMAN | | | | | proteasome (prosome, macropain) subunit, alpha type, 5 | | | | | | -0.07 | | | | | | | 0.43 | | | | | | |  |
| PSA6_HUMAN | | | | | proteasome (prosome, macropain) subunit, alpha type, 6 | | | | | | 0.05 | | | | | | | 0.43 | | | | | | |  |
| PSA7_HUMAN | | | | | proteasome (prosome, macropain) subunit, alpha type, 7 | | | | | | 0 | | | | | | | 0.98 | | | | | | |  |
| PSB6_HUMAN | | | | | proteasome (prosome, macropain) subunit, beta type, 6 | | | | | | 0.04 | | | | | | | 0.57 | | | | | | |  |
| PSMD1_HUMAN | | | | | proteasome (prosome, macropain) 26S subunit, non-ATPase, 1 | | | | | | -0.02 | | | | | | | 0.90 | | | | | | |  |
| PSMD2_HUMAN | | | | | proteasome (prosome, macropain) 26S subunit, non-ATPase, 2 | | | | | | 0 | | | | | | | 0.99 | | | | | | |  |
| PSMD3_HUMAN | | | | | proteasome (prosome, macropain) 26S subunit, non-ATPase, 3 | | | | | | 0.17 | | | | | | | 0.00 | | | | | | |  |
| PSME1_HUMAN | | | | | proteasome (prosome, macropain) activator subunit 1 (PA28 alpha) | | | | | | 0.02 | | | | | | | 0.91 | | | | | | |  |
| PTBP1_HUMAN | | | | | polypyrimidine tract binding protein 1 | | | | | | -0.07 | | | | | | | 0.33 | | | | | | |  |
| ASC_HUMAN | | | | | PYD and CARD domain containing | | | | | | 0.04 | | | | | | | 0.58 | | | | | | |  |
| RAB10_HUMAN | | | | | RAB10, member RAS oncogene family | | | | | | 0.03 | | | | | | | 0.68 | | | | | | |  |
| RB11A_HUMAN | | | | | RAB11A, member RAS oncogene family | | | | | | 0.13 | | | | | | | 0.19 | | | | | | |  |
| RAB5C_HUMAN | | | | | RAB5C, member RAS oncogene family | | | | | | 0.12 | | | | | | | 0.14 | | | | | | |  |
| RAC1_HUMAN | | | | | ras-related C3 botulinum toxin substrate 1 (rho family, small GTP binding protein Rac1) | | | | | | 0.08 | | | | | | | 0.33 | | | | | | |  |
| RAN_HUMAN | | | | | RAN, member RAS oncogene family | | | | | | -0.02 | | | | | | | 0.86 | | | | | | |  |
| SYRC_HUMAN | | | | | arginyl-tRNA synthetase | | | | | | 0.14 | | | | | | | 0.13 | | | | | | |  |
| RNAS7_HUMAN | | | | | ribonuclease, RNase A family, 7 | | | | | | -0.03 | | | | | | | 0.77 | | | | | | |  |
| RINI_HUMAN | | | | | ribonuclease/angiogenin inhibitor 1 | | | | | | -0.04 | | | | | | | 0.49 | | | | | | |  |
| RL10A_HUMAN | | | | | ribosomal protein L10a | | | | | | -0.04 | | | | | | | 0.71 | | | | | | |  |
| RL11_HUMAN | | | | | ribosomal protein L11 | | | | | | -0.15 | | | | | | | 0.09 | | | | | | |  |
| RL12_HUMAN | | | | | ribosomal protein L12 | | | | | | -0.04 | | | | | | | 0.61 | | | | | | |  |
| RL13_HUMAN | | | | | ribosomal protein L13 | | | | | | 0.06 | | | | | | | 0.41 | | | | | | |  |
| RL17_HUMAN | | | | | ribosomal protein L17 | | | | | | 0.03 | | | | | | | 0.74 | | | | | | |  |
| RL18_HUMAN | | | | | ribosomal protein L18 | | | | | | 0.01 | | | | | | | 0.89 | | | | | | |  |
| RL22_HUMAN | | | | | ribosomal protein L22 | | | | | | 0.21 | | | | | | | 0.03 | | | | | | |  |
| RL23_HUMAN | | | | | ribosomal protein L23 | | | | | | 0.01 | | | | | | | 0.82 | | | | | | |  |
| RL23A_HUMAN | | | | | ribosomal protein L23a | | | | | | 0.11 | | | | | | | 0.21 | | | | | | |  |
| RL28_HUMAN | | | | | ribosomal protein L28 | | | | | | 0.1 | | | | | | | 0.42 | | | | | | |  |
| RL3_HUMAN | | | | | ribosomal protein L3 | | | | | | 0.16 | | | | | | | 0.09 | | | | | | |  |
| RL30_HUMAN | | | ribosomal protein L30 | | | | | | 0.13 | | | | | | 0.25 | | | | | | | |  |  |  |
| RL34_HUMAN | | | ribosomal protein L34 | | | | | | -0.03 | | | | | | 0.77 | | | | | | | |  |  |  |
| RL4_HUMAN | | | ribosomal protein L4 | | | | | | 0.17 | | | | | | 0.01 | | | | | | | |  |  |  |
| RL5_HUMAN | | | ribosomal protein L5 | | | | | | 0.02 | | | | | | 0.72 | | | | | | | |  |  |  |
| RL6_HUMAN | | | ribosomal protein L6 | | | | | | 0.09 | | | | | | 0.33 | | | | | | | |  |  |  |
| RL7_HUMAN | | | ribosomal protein L7 | | | | | | 0.04 | | | | | | 0.53 | | | | | | | |  |  |  |
| RL7A_HUMAN | | | ribosomal protein L7a | | | | | | -0.05 | | | | | | 0.53 | | | | | | | |  |  |  |
| RL8_HUMAN | | | ribosomal protein L8 | | | | | | 0.16 | | | | | | 0.15 | | | | | | | |  |  |  |
| RL9_HUMAN | | | ribosomal protein L9 | | | | | | 0 | | | | | | 1.00 | | | | | | | |  |  |  |
| RLA0_HUMAN | | | ribosomal protein, large, P0 | | | | | | 0.04 | | | | | | 0.29 | | | | | | | |  |  |  |
| RLA2_HUMAN | | | ribosomal protein, large, P2 | | | | | | 0 | | | | | | 0.97 | | | | | | | |  |  |  |
| RPN1_HUMAN | | | ribophorin I | | | | | | -0.01 | | | | | | 0.91 | | | | | | | |  |  |  |
| RS10_HUMAN | | | ribosomal protein S10 | | | | | | 0.01 | | | | | | 0.90 | | | | | | | |  |  |  |
| RS11_HUMAN | | | ribosomal protein S11 | | | | | | 0.13 | | | | | | 0.11 | | | | | | | |  |  |  |
| RS13_HUMAN | | | ribosomal protein S13 | | | | | | -0.13 | | | | | | 0.09 | | | | | | | |  |  |  |
| RS14_HUMAN | | | ribosomal protein S14 | | | | | | 0.14 | | | | | | 0.22 | | | | | | | |  |  |  |
| RS15A_HUMAN | | | ribosomal protein S15a | | | | | | 0.1 | | | | | | 0.34 | | | | | | | |  |  |  |
| RS16_HUMAN | | | ribosomal protein S16 | | | | | | -0.06 | | | | | | 0.41 | | | | | | | |  |  |  |
| RS18_HUMAN | | | ribosomal protein S18 | | | | | | -0.16 | | | | | | 0.15 | | | | | | | |  |  |  |
| RS2_HUMAN | | | ribosomal protein S2 | | | | | | 0.05 | | | | | | 0.58 | | | | | | | |  |  |  |
| RS25_HUMAN | | | ribosomal protein S25 | | | | | | -0.21 | | | | | | 0.07 | | | | | | | |  |  |  |
| RS26_HUMAN | | | ribosomal protein S26 | | | | | | 0.07 | | | | | | 0.53 | | | | | | | |  |  |  |
| RS27_HUMAN | | | ribosomal protein S27 | | | | | | 0.21 | | | | | | 0.02 | | | | | | | |  |  |  |
| RS27A_HUMAN | | | ribosomal protein S27a | | | | | | 0.05 | | | | | | 0.36 | | | | | | | |  |  |  |
| RS3_HUMAN | | | ribosomal protein S3 | | | | | | -0.03 | | | | | | 0.60 | | | | | | | |  |  |  |
| RS3A_HUMAN | | | ribosomal protein S3A | | | | | | 0.03 | | | | | | 0.50 | | | | | | | |  |  |  |
| RS4X_HUMAN | | | ribosomal protein S4, X-linked | | | | | | -0.04 | | | | | | 0.39 | | | | | | | |  |  |  |
| RS5_HUMAN | | | ribosomal protein S5 | | | | | | -0.12 | | | | | | 0.04 | | | | | | | |  |  |  |
| RS6_HUMAN | | | ribosomal protein S6 | | | | | | 0.15 | | | | | | 0.10 | | | | | | | |  |  |  |
| RS7_HUMAN | | | ribosomal protein S7 | | | | | | -0.03 | | | | | | 0.80 | | | | | | | |  |  |  |
| RS8_HUMAN | | | | ribosomal protein S8 | | | | | | 0.03 | | | | | | 0.49 | | | | | | | |  |  |
| RS9_HUMAN | | | | ribosomal protein S9 | | | | | | -0.03 | | | | | | 0.74 | | | | | | | |  |  |
| RSSA_HUMAN | | | | ribosomal protein SA | | | | | | 0.04 | | | | | | 0.42 | | | | | | | |  |  |
| S10AA_HUMAN | | | | S100 calcium binding protein A10 | | | | | | -0.06 | | | | | | 0.42 | | | | | | | |  |  |
| S10AB_HUMAN | | | | S100 calcium binding protein A11 | | | | | | 0.03 | | | | | | 0.66 | | | | | | | |  |  |
| S10AE_HUMAN | | | | S100 calcium binding protein A14 | | | | | | 0.09 | | | | | | 0.17 | | | | | | | |  |  |
| S10AG_HUMAN | | | | S100 calcium binding protein A16 | | | | | | 0.05 | | | | | | 0.64 | | | | | | | |  |  |
| S10A2_HUMAN | | | | S100 calcium binding protein A2 | | | | | | 0.04 | | | | | | 0.61 | | | | | | | |  |  |
| S10A6_HUMAN | | | | S100 calcium binding protein A6 | | | | | | -0.03 | | | | | | 0.71 | | | | | | | |  |  |
| S10A7_HUMAN | | | | S100 calcium binding protein A7 | | | | | | 0.06 | | | | | | 0.56 | | | | | | | |  |  |
| S10A8_HUMAN | | | | S100 calcium binding protein A8 | | | | | | -0.11 | | | | | | 0.28 | | | | | | | |  |  |
| S10A9_HUMAN | | | | S100 calcium binding protein A9 | | | | | | -0.12 | | | | | | 0.17 | | | | | | | |  |  |
| SBSN_HUMAN | | | | suprabasin | | | | | | -0.02 | | | | | | 0.87 | | | | | | | |  |  |
| SCEL_HUMAN | | | | sciellin | | | | | | 0.04 | | | | | | 0.75 | | | | | | | |  |  |
| SC61B_HUMAN | | | | Sec61 beta subunit | | | | | | 0.13 | | | | | | 0.29 | | | | | | | |  |  |
| PAIRB_HUMAN | | | | SERPINE1 mRNA binding protein 1 | | | | | | 0.01 | | | | | | 0.92 | | | | | | | |  |  |
| SPB3_HUMAN | | | | serpin peptidase inhibitor, clade B (ovalbumin), member 3 | | | | | | 0.02 | | | | | | 0.75 | | | | | | | |  |  |
| SPB5_HUMAN | | | | serpin peptidase inhibitor, clade B (ovalbumin), member 5 | | | | | | 0.01 | | | | | | 0.71 | | | | | | | |  |  |
| SET_HUMAN | | | | SET nuclear proto-oncogene | | | | | | -0.03 | | | | | | 0.82 | | | | | | | |  |  |
| 1433S_HUMAN | | | | stratifin | | | | | | 0.01 | | | | | | 0.77 | | | | | | | |  |  |
| SFPQ_HUMAN | | | | splicing factor proline/glutamine-rich | | | | | | -0.01 | | | | | | 0.90 | | | | | | | |  |  |
| ADT2_HUMAN | | | | solute carrier family 25 (mitochondrial carrier; adenine nucleotide translocator), member 5 | | | | | | 0.02 | | | | | | 0.80 | | | | | | | |  |  |
| 4F2_HUMAN | | | | solute carrier family 3 (amino acid transporter heavy chain), member 2 | | | | | | -0.05 | | | | | | 0.32 | | | | | | | |  |  |
| LAT1_HUMAN | | | | solute carrier family 7 (amino acid transporter light chain, L system), member 5 | | | | | | 0.17 | | | | | | 0.10 | | | | | | | |  |  |
| SLPI_HUMAN | | | | secretory leukocyte peptidase inhibitor | | | | | | 0.08 | | | | | | 0.43 | | | | | | | |  |  |
| SND1_HUMAN | | | | staphylococcal nuclease and tudor domain containing 1 | | | | | | 0.02 | | | | | | 0.87 | | | | | | | |  |  |
| SODC_HUMAN | | | | superoxide dismutase 1, soluble | | | | | | 0.03 | | | | | | 0.66 | | | | | | | |  |  |
| SPR1B_HUMAN | | | | small proline-rich protein 1B | | | | | | 0.04 | | | | | | 0.57 | | | | | | | |  |  |
| SPR2D_HUMAN | | | | small proline-rich protein 2D | | | | | | 0.15 | | | | | | 0.16 | | | | | | | |  |  |
| SPR2F_HUMAN | | | | small proline-rich protein 2F | | | | | | 0.16 | | | | | | 0.19 | | | | | | | |  |  |
| SQRD_HUMAN | | | | | | sulfide quinone reductase-like (yeast) | | | | | | 0.19 | | | | | | | 0.07 | | | |  |  |  |
| SSRD_HUMAN | | | | | | signal sequence receptor, delta | | | | | | 0.04 | | | | | | | 0.71 | | | |  |  |  |
| STIP1_HUMAN | | | | | | stress-induced phosphoprotein 1 | | | | | | 0.1 | | | | | | | 0.38 | | | |  |  |  |
| HNRPQ_HUMAN | | | | | | synaptotagmin binding, cytoplasmic RNA interacting protein | | | | | | -0.04 | | | | | | | 0.74 | | | |  |  |  |
| TAGL2_HUMAN | | | | | | transgelin 2 | | | | | | -0.02 | | | | | | | 0.75 | | | |  |  |  |
| TALDO_HUMAN | | | | | | transaldolase 1 | | | | | | 0.06 | | | | | | | 0.41 | | | |  |  |  |
| TCPA_HUMAN | | | | | | t-complex 1 | | | | | | -0.15 | | | | | | | 0.21 | | | |  |  |  |
| TGM1_HUMAN | | | | | | transglutaminase 1 | | | | | | -0.07 | | | | | | | 0.52 | | | |  |  |  |
| TGM3_HUMAN | | | | | | transglutaminase 3 | | | | | | -0.07 | | | | | | | 0.40 | | | |  |  |  |
| TKT_HUMAN | | | | | | transketolase | | | | | | -0.02 | | | | | | | 0.66 | | | |  |  |  |
| TLN1_HUMAN | | | | | | talin 1 | | | | | | -0.06 | | | | | | | 0.72 | | | |  |  |  |
| TB182_HUMAN | | | | | | tankyrase 1 binding protein 1, 182kDa | | | | | | 0.05 | | | | | | | 0.79 | | | |  |  |  |
| TPIS_HUMAN | | | | | | triosephosphate isomerase 1 | | | | | | 0.02 | | | | | | | 0.82 | | | |  |  |  |
| TPM4_HUMAN | | | | | | tropomyosin 4 | | | | | | -0.04 | | | | | | | 0.61 | | | |  |  |  |
| TRI29_HUMAN | | | | | | tripartite motif containing 29 | | | | | | 0.08 | | | | | | | 0.02 | | | |  |  |  |
| TBA4B_HUMAN | | | | | | tubulin, alpha 4b (pseudogene) | | | | | | -0.03 | | | | | | | 0.54 | | | |  |  |  |
| TBB5_HUMAN | | | | | | tubulin, beta class I | | | | | | -0.09 | | | | | | | 0.17 | | | |  |  |  |
| EFTU_HUMAN | | | | | | Tu translation elongation factor, mitochondrial | | | | | | -0.01 | | | | | | | 0.96 | | | |  |  |  |
| THIO_HUMAN | | | | | | thioredoxin | | | | | | 0.16 | | | | | | | 0.01 | | | |  |  |  |
| TXD17_HUMAN | | | | | | thioredoxin domain containing 17 | | | | | | 0.06 | | | | | | | 0.48 | | | |  |  |  |
| TYPH_HUMAN | | | | | | thymidine phosphorylase | | | | | | 0.04 | | | | | | | 0.59 | | | |  |  |  |
| UBA1_HUMAN | | | | | | ubiquitin-like modifier activating enzyme 1 | | | | | | -0.06 | | | | | | | 0.51 | | | |  |  |  |
| QCR2_HUMAN | | | | | | ubiquinol-cytochrome c reductase core protein II | | | | | | 0.07 | | | | | | | 0.58 | | | |  |  |  |
| VAT1_HUMAN | | | | | | vesicle amine transport 1 | | | | | | 0.06 | | | | | | | 0.59 | | | |  |  |  |
| VINC_HUMAN | | | | | | vinculin | | | | | | 0 | | | | | | | 0.99 | | | |  |  |  |
| TERA_HUMAN | | | | | | valosin containing protein | | | | | | 0.03 | | | | | | | 0.58 | | | |  |  |  |
| VDAC1_HUMAN | | | | | | voltage-dependent anion channel 1 | | | | | | 0.03 | | | | | | | 0.61 | | | |  |  |  |
| VDAC2_HUMAN | | | | | | voltage-dependent anion channel 2 | | | | | | 0.09 | | | | | | | 0.16 | | | |  |  |  |
| VIME_HUMAN | | | | | | vimentin | | | | | | -0.32 | | | | | | | 0.02 | | | |  |  |  |
| WDR1_HUMAN | | | | | | WD repeat domain 1 | | | | | | -0.13 | | | | | | | 0.47 | | | |  |  |  |
| YBOX1_HUMAN | | | | | | Y box binding protein 1 | | | | | | 0.13 | | | | | | | 0.38 | | | | | | |
| ZN185_HUMAN | | | | | | zinc finger protein 185 (LIM domain) | | | | | | -0.02 | | | | | | | 0.86 | | | | | | |

| **Uniprot ID** | **Protein name** | **Symbol** | **Mean difference (LOG_10_)** | **P-value** |
| --- | --- | --- | --- | --- |
| ANXA3_HUMAN | Annexin A3 | ANXA3 | 0.17 | 0.0225 |
| CATL2_HUMAN | Cathepsin L2 | CTSV | 0.15 | 0.048 |
| CBPA4_HUMAN | Carboxypeptidase A4 | CPA4 | 0.25 | 0.0365 |
| COF1_HUMAN | Cofilin-1 | CFL1 | 0.11 | 0.0331 |
| FILA_HUMAN | Filaggrin | FLG | -0.46 | 2.00E-04 |
| KLK7_HUMAN | Kallikrein-7 | KLK7 | 0.34 | 0.02 |
| LY6D_HUMAN | Lymphocyte antigen 6D | LY6D | 0.14 | 0.0374 |
| PPIA_HUMAN | Cyclophilin A | PPIA | -0.06 | 0.0039 |
| PSMD3_HUMAN | 26S proteasome regulatory subunit 3 | PSMD3 | 0.17 | 0.0031 |
| RL22_HUMAN | 60S ribosomal protein L22 | RPL22 | 0.21 | 0.0312 |
| RL4_HUMAN | 60S ribosomal protein L4 | RPL4 | 0.17 | 0.0137 |
| RS27_HUMAN | 40S ribosomal protein S27 | RPS27 | 0.21 | 0.0201 |
| RS5_HUMAN | 40S ribosomal protein S5 | RPS5 | -0.12 | 0.0425 |
| TCPZ_HUMAN | T-complex protein 1 subunit zeta | CCT6A | -0.11 | 0.0143 |
| THIO_HUMAN | Thioredoxin | TXN | 0.16 | 0.0111 |
|  |  |  |  |  |
| TRI29_HUMAN | Tripartite motif-containing protein 29 | TRIM29 | 0.08 | 0.018 |
| VIME_HUMAN | Vimentin | VIM | -0.32 | 0.024 |

**Table E2: List of significantly differentially expressed proteins following filaggrin knockdown**

**Table E3: Top 5 disease and disorders predicted by IPA**

| **IPA Diseases & disorders** | **(p-value)** | **Molecules** |
| --- | --- | --- |
| Immunological disease | 2.15E-06 - 3.58E-02 | ↑ANXA3, ↑CFL1, ↑CSTV, ↓FLG, ↑KLK7, ↓PPIA, ↓VIM, ↑TXN, ↑LY6D |
| Dermatological disease | 2.42E-05 - 3.99E-02 | ↑CFL1, ↑CSTV, ↓FLG, ↑KLK7, ↑ANXA3, ↓VIM |
| Inflammatory disease | 2.42E-05 - 3.66E-03 | ↑ANXA3, ↑CFL1, ↑CSTV, ↓FLG, ↑KLK7, ↓PPIA, ↓VIM |
| Inflammatory response | 2.42E-05 - 3.86E-04 | ↑ANXA3, ↑CFL1, ↑CSTV, ↓FLG, ↑KLK7, ↓PPIA, ↓VIM, ↑TXN |
| Neurological disease | 1.84E-05 - 4.96E-05 | ↑CSTV, ↓PPIA, ↓VIM, ↑TXN |

Ingenuity pathway analysis (IPA)

**Table E4: List of functional annotations associated with the top 5 disease** **and disorder categories**

| **Categories** | **Function** | | | | | **P-value** | | | | | **Molecules** | | | | | |
| --- | --- | --- | --- | --- | --- | --- | --- | --- | --- | --- | --- | --- | --- | --- | --- | --- |
| Cellular movement | migration of smooth muscle cells | | | | | 3.05E-04 | | | | | CTSV,PPIA,TXN | | | | | |
| Cellular movement | chemotaxis of inflammatory leukocytes | | | | | 8.46E-04 | | | | | PPIA | | | | | |
| Cellular movement | scattering of neuronal progenitor cells | | | | | 8.46E-04 | | | | | CFL1 | | | | | |
| Cellular movement | cell rolling of squamous cell carcinoma cell lines | | | | | 1.69E-03 | | | | | LY6D | | | | | |
| Cellular movement | migration of AT2 cells | | | | | 1.69E-03 | | | | | VIM | | | | | |
| Cellular movement | migration of podocytes | | | | | 1.69E-03 | | | | | CTSV | | | | | |
| Cellular movement | delay in transmigration of smooth muscle cells | | | | | 2.54E-03 | | | | | CTSV | | | | | |
| Cellular movement | migration of cells | | | | | 3.06E-03 | | | | | ANXA3,CFL1,CTSV,LY6D,PPIA,TXN,VIM | | | | | |
| Cellular movement | migration of vascular smooth muscle cells | | | | | 3.63E-03 | | | | | PPIA,TXN | | | | | |
| Cellular movement | cell movement of eosinophils | | | | | 4.10E-03 | | | | | PPIA,TXN | | | | | |
| Cellular movement | migration of inflammatory leukocytes | | | | | 4.22E-03 | | | | | PPIA | | | | | |
| Cellular movement | recruitment of leukemia cell lines | | | | | 4.22E-03 | | | | | VIM | | | | | |
| Cellular movement | invasion of thyroid tumor cell lines | | | | | 7.59E-03 | | | | | VIM | | | | | |
| Cellular movement | cell movement of monocytes | | | | | 1.00E-02 | | | | | CTSV,PPIA | | | | | |
| Cellular movement | chemotaxis of peripheral blood monocytes | | | | | 1.18E-02 | | | | | PPIA | | | | | |
| Cellular movement | cell movement of embryonic stem cells | | | | | 1.26E-02 | | | | | CFL1 | | | | | |
| Cellular movement | infiltration by mast cells | | | | | 1.35E-02 | | | | | PPIA | | | | | |
| Cellular movement | cell movement of myeloid cells | | | | | 1.80E-02 | | | | | CTSV,PPIA,TXN | | | | | |
| Cellular movement | cell movement of phagocytes | | | | | 1.91E-02 | | | | | CTSV,PPIA,TXN | | | | | |
| Cellular movement | migration of tumor cells | | | | | 1.98E-02 | | | | | LY6D,VIM | | | | | |
| Cellular movement | chemotaxis of embryonic cell lines | | | | | 2.01E-02 | | | | | PPIA | | | | | |
| Cellular movement | chemotaxis of epithelial cell lines | | | | | 2.01E-02 | | | | | PPIA | | | | | |
| Cellular movement | migration of melanoma cells | | | | | 2.01E-02 | | | | | VIM | | | | | |
| Cellular movement | chemotaxis of kidney cell lines | | | | | 2.51E-02 | | | | | PPIA | | | | | |
| Cellular movement | migration of phagocytes | | | | | 2.89E-02 | | | | | CTSV,TXN | | | | | |
| Cellular movement | migration of eosinophils | | | | | 2.92E-02 | | | | | TXN | | | | | |
| Cellular movement | transmigration of T lymphocytes | | | | | 3.17E-02 | | | | | CTSV | | | | | |
| Cellular movement | transmigration of monocytes | | | | | 3.33E-02 | | | | | CTSV | | | | | |
| Cellular movement | migration of endothelial cells | | | | | 4.06E-02 | | | | | ANXA3,VIM | | | | | |
| Cellular movement | migration of neural crest cells | | | | | 4.31E-02 | | | | | CFL1 | | | | | |
| Cellular movement | | | | chemotaxis of leukocyte cell lines | | | | | 4.39E-02 | | | | | TXN | |  |
| Cellular movement | | | | recruitment of cells | | | | | 4.76E-02 | | | | | TXN,VIM | |  |
| Cellular movement | | | | migration of breast cell lines | | | | | 4.80E-02 | | | | | VIM | |  |
| Cellular movement | | | | chemotaxis of leukocytes | | | | | 4.86E-02 | | | | | PPIA,TXN | |  |
| Cellular movement | | | | infiltration by eosinophils | | | | | 4.96E-02 | | | | | PPIA | |  |
| Cellular Development | | | | differentiation of keratinocytes | | | | | 8.02E-03 | | | | | CTSV,FLG | |  |
| Cellular Development | | | | differentiation of cells | | | | | 2.94E-02 | | | | | CFL1,CTSV,FLG,PPIA,RPL22,VIM | |  |
| Cellular Development | | | | development of astrocytes | | | | | 1.26E-02 | | | | | VIM | |  |
| Cellular Development | | | | differentiation of alpha-beta T lymphocytes | | | | | 1.93E-02 | | | | | RPL22 | |  |
| Cellular Development | | | | differentiation of epidermis | | | | | 1.93E-02 | | | | | FLG | |  |
| Cellular Development | | | | differentiation of germ cell tumor cell lines | | | | | 4.07E-02 | | | | | PPIA | |  |
| Cellular Development, growth and proliferation | | | | proliferation of monocytes | | | | | 3.94E-04 | | | | | PPIA,TXN | |  |
| Cellular Development, growth and proliferation | | | | proliferation of basal keratinocytes | | | | | 8.46E-04 | | | | | CTSV | |  |
| Cellular Development, growth and proliferation | | | | proliferation of islet cell tumor | | | | | 1.69E-03 | | | | | CTSV | |  |
| Cellular Development, growth and proliferation | | | | arrest in development of double-negative T lymphocyte | | | | | 4.22E-03 | | | | | RPL22 | |  |
| Cellular Development, growth and proliferation | | | | arrest in growth of cervical cancer cell lines | | | | | 4.22E-03 | | | | | TXN | |  |
| Cellular Development, growth and proliferation | | | | proliferation of melanocytes | | | | | 2.01E-02 | | | | | CTSV | |  |
| Cellular Development, growth and proliferation | | | | proliferation of neuronal cells | | | | | 2.28E-02 | | | | | CFL1,TXN,VIM | |  |
| Cellular Development, growth and proliferation | | | | proliferation of neuronal progenitor cells | | | | | 4.47E-02 | | | | | CFL1 | |  |
| Cellular Development, growth and proliferation | | | | proliferation of vascular smooth muscle cells | | | | | 8.60E-03 | | | | | PPIA,TXN | |  |
| Cell Morphology | | | | morphology of nuclear matrix | | | | | 8.46E-04 | | | | | VIM | |  |
| Cell Morphology | | | | polarization of neural crest cells | | | | | 8.46E-04 | | | | | CFL1 | |  |
| Cell Morphology | | | | vacuolation of melanocytes | | | | | 8.46E-04 | | | | | CTSV | |  |
| Cell Morphology | | | | formation of microtentacles | | | | | 1.69E-03 | | | | | VIM | |  |
| Cell Morphology | | | | contraction of fibroblast cell lines | | | | | 3.38E-03 | | | | | VIM | |  |
| Cell Morphology | | | | morphology of thyroid tumor cell lines | | | | | 5.91E-03 | | | | | VIM | |  |
| Cell Morphology | | | | polarization of M2 macrophages | | | | | 5.91E-03 | | | | | TXN | |  |
| Cell Morphology | | | | cell spreading of embryonic cell lines | | | | | 9.27E-03 | | | | | VIM | |  |
| Cell Morphology | | | | polarization of cells | | | | | 1.01E-02 | | | | | CFL1,TXN | |  |
| Cell Morphology | | | | cell spreading of epithelial cell lines | | | | | 1.26E-02 | | | | | VIM | |  |
| Cell Morphology | | | | morphology of carcinoma cell lines | | | | | 1.68E-02 | | | | | VIM | |  |
| Cell Morphology | | | | cell spreading of endothelial cells | | | | | 1.93E-02 | | | | | VIM | |  |
| Cell Morphology | | | cell spreading of kidney cell lines | | | | | 1.93E-02 | | | | | VIM | | |  |
| Cell Morphology | | | morphology of astrocytes | | | | | 3.74E-02 | | | | | VIM | | |  |
| Cell Morphology | | | cell spreading of fibroblast cell lines | | | | | 4.23E-02 | | | | | VIM | | |  |
| Cell Death and Survival | | | cell death of fibroblast cell lines | | | | | 8.36E-04 | | | | | CTSV,TRIM29,TXN,VIM | | |  |
| Cell Death and Survival | | | cell death of fibrosarcoma cells | | | | | 8.46E-04 | | | | | TXN | | |  |
| Cell Death and Survival | | | cytotoxicity of cyclosporin A | | | | | 1.69E-03 | | | | | PPIA | | |  |
| Cell Death and Survival | | | cytotoxicity of eosinophils | | | | | 4.22E-03 | | | | | TXN | | |  |
| Cell Death and Survival | | | apoptosis of lymphoid cells | | | | | 6.75E-03 | | | | | TXN | | |  |
| Cell Death and Survival | | | apoptosis of chronic lymphocytic leukemia B cells | | | | | 8.43E-03 | | | | | TXN | | |  |
| Cell Death and Survival | | | cell death of neuroblastoma cell lines | | | | | 1.26E-02 | | | | | CCT6A,TXN | | |  |
| Cell Death and Survival | | | apoptosis of endothelial cells | | | | | 1.29E-02 | | | | | PPIA,TXN | | |  |
| Cell Death and Survival | | | apoptosis of thymoma cell lines | | | | | 1.43E-02 | | | | | TXN | | |  |
| Cell Death and Survival | | | necrosis of prostate cancer cell lines | | | | | 1.51E-02 | | | | | PPIA,TXN | | |  |
| Cell Death and Survival | | | apoptosis of colon cancer cell lines | | | | | 2.01E-02 | | | | | PPIA,TRIM29 | | |  |
| Cell Death and Survival | | | apoptosis of osteoclasts | | | | | 2.09E-02 | | | | | VIM | | |  |
| Cell Death and Survival | | | cytotoxicity | | | | | 2.17E-02 | | | | | PPIA,TXN | | |  |
| Cell Death and Survival | | | cell death of bladder cancer cell lines | | | | | 2.67E-02 | | | | | TXN | | |  |
| Cell Death and Survival | | | apoptosis of cervical cancer cell lines | | | | | 2.82E-02 | | | | | PPIA,TXN | | |  |
| Cell Death and Survival | | | apoptosis of lung cell lines | | | | | 2.92E-02 | | | | | TXN | | |  |
| Cell Death and Survival | | | neurodegeneration of Purkinje cells | | | | | 3.41E-02 | | | | | CTSV | | |  |
| Cell Death and Survival | | | apoptosis of fibroblast cell lines | | | | | 4.14E-02 | | | | | TRIM29,VIM | | |  |
| Cell Death and Survival | | | cell viability of embryonic cell lines | | | | | 4.96E-02 | | | | | PPIA | | |  |
| Immunological Disease | | allergy | | | | | 2.15E-06 | | | | | ANXA3,CFL1,CTSV,FLG,KLK7,PPIA | | | | |
| Immunological Disease | | immediate hypersensitivity | | | | | 1.29E-05 | | | | | CFL1,CTSV,FLG,KLK7,PPIA | | | | |
| Immunological Disease | | systemic autoimmune syndrome | | | | | 2.51E-02 | | | | | CTSV,LY6D,TXN,VIM | | | | |
| Immunological Disease | | hairy-cell leukemia | | | | | 2.99E-02 | | | | | CFL1,FLG | | | | |
| Immunological Disease, Inflammatory disease | | allergic rhinitis | | | | | 3.58E-02 | | | | | FLG | | | | |
| Immunological Disease, Inflammatory response | | allergic pulmonary eosinophilia | | | | | 2.76E-02 | | | | | ANXA3 | | | | |
| Dermatological Disease, Immunological Disease, Inflammatory Disease, Inflammatory Response | | atopic dermatitis | | | | | 1.09E-04 | | | | | CFL1,CTSV,FLG,KLK7 | | | | |
| Dermatological Disease, Immunological Disease, Inflammatory Response | | susceptibility to atopic dermatitis type 2 | | | | | 8.46E-04 | | | | | FLG | | | | |
| Dermatological Disease, Immunological Disease, Inflammatory Response | | advanced stage atopic dermatitis | | | | | 7.59E-03 | | | | | FLG | | | | |
| Dermatological Disease, Inflammatory Disease, Inflammatory Response | | folliculitis | | | | | 5.07E-03 | | | | | CTSV | | | | |
| Dermatological Disease, Inflammatory Disease, Inflammatory Response | | inflammation of dermis | | | | | 2.54E-03 | | | | | KLK7 | | | | |
| Dermatological Disease, Inflammatory Disease, Inflammatory Response | | inflammation of skin component | | | | | 2.42E-05 | | | | | CTSV,KLK7 | | | | |
| Dermatological Diseases | | psoriasis | | | | | 2.45E-04 | | | | | ANXA3,CFL1,CTSV,FLG,KLK7 | | | | |
| Dermatological Diseases | | acanthosis of epithelial tissue | | | | | 8.46E-04 | | | | | CTSV | | | | |
| Dermatological Diseases | | ichthyosis vulgaris | | | | | 8.46E-04 | | | | | FLG | | | | |
| Dermatological Diseases | | severe pruritus | | | | | 8.46E-04 | | | | | KLK7 | | | | |
| Dermatological Diseases | | Carney complex type 1 | | | | | 1.69E-03 | | | | | VIM | | | | |
| Dermatological Diseases | | hyperkeratosis | | | | | 2.87E-03 | | | | | CTSV,KLK7 | | | | |
| Dermatological Diseases | | hyperplasia of sebaceous glands | | | | | 4.22E-03 | | | | | CTSV | | | | |
| Dermatological Diseases | | scratch lesion | | | | | 5.07E-03 | | | | | VIM | | | | |
| Dermatological Diseases | | active stage psoriasis | | | | | 1.43E-02 | | | | | ANXA3 | | | | |
| Dermatological Diseases | | senile lentigo | | | | | 3.99E-02 | | | | | FLG | | | | |
| Inflammatory Disease, Neurological disease | | neuroinflammation of brain | | | | | 5.07E-03 | | | | | VIM | | | | |
| Inflammatory Disease | | blepharitis | | | | | 1.01E-02 | | | | | PPIA | | | | |
| Inflammatory Disease, | | keratitis | | | | | 3.09E-02 | | | | | CTSV | | | | |
| Inflammatory Disease | | insulitis | | | | | 3.66E-02 | | | | | CTSV | | | | |
| Inflammatory Response | | inflammation of organ | | | | | 1.32E-04 | | | | | ANXA3,CFL1,CTSV,FLG,KLK7,PPIA,VIM | | | | |
| Inflammatory Response | | proliferation of monocytes | | | | | 3.94E-04 | | | | | PPIA,TXN | | | | |
| Inflammatory Response | | inflammation of secretory structure | | | | | 8.06E-04 | | | | | CTSV,PPIA | | | | |
| Inflammatory Response | | | | | chemotaxis of inflammatory leukocytes | | | | | 8.46E-04 | | | | | PPIA |  |
| Inflammatory Response | | | | | inflammation of footpad | | | | | 5.91E-03 | | | | | PPIA |  |
| Inflammatory Response | | | | | alternative complement pathway | | | | | 7.59E-03 | | | | | TXN |  |
| Inflammatory Response | | | | | cell movement of monocytes | | | | | 1.00E-02 | | | | | CTSV,PPIA |  |
| Inflammatory Response | | | | | chemotaxis of peripheral blood monocytes | | | | | 1.18E-02 | | | | | PPIA |  |
| Inflammatory Response | | | | | infiltration by mast cells | | | | | 1.35E-02 | | | | | PPIA |  |
| Inflammatory Response | | | | | classical complement pathway | | | | | 1.43E-02 | | | | | TXN |  |
| Inflammatory Response | | | | | inflammation of body region | | | | | 1.65E-02 | | | | | ANXA3,CTSV,PPIA,VIM |  |
| Inflammatory Response | | | | | inflammation of salivary gland | | | | | 1.85E-02 | | | | | CTSV |  |
| Inflammatory Response | | | | | cell movement of phagocytes | | | | | 1.91E-02 | | | | | CTSV,PPIA,TXN |  |
| Inflammatory Response | | | | | inflammation of pancreas | | | | | 2.34E-02 | | | | | PPIA |  |
| Inflammatory Response | | | | | degranulation of neutrophils | | | | | 2.51E-02 | | | | | ANXA3 |  |
| Inflammatory Response | | | | | phagocytosis of leukemia cell lines | | | | | 2.51E-02 | | | | | VIM |  |
| Inflammatory Response | | | | | phagocytosis of cells | | | | | 2.67E-02 | | | | | ANXA3,VIM |  |
| Inflammatory Response | | | | | antimicrobial response | | | | | 2.75E-02 | | | | | ANXA3,PPIA |  |
| Inflammatory Response | | | | | migration of phagocytes | | | | | 2.89E-02 | | | | | CTSV,TXN |  |
| Inflammatory Response | | | | | transmigration of monocytes | | | | | 3.33E-02 | | | | | CTSV |  |
| Inflammatory Response | | | | | inflammation of heart | | | | | 3.50E-02 | | | | | PPIA |  |
| Inflammatory Response | | | | | chemotaxis of leukocytes | | | | | 4.86E-02 | | | | | PPIA,TXN |  |
| Neurological Disease | | | | | atrophy of brain | | | | | 1.84E-04 | | | | | CTSV,VIM |  |
| Neurological Disease | | | | | neurodegeneration of cerebellum | | | | | 7.12E-04 | | | | | CTSV,VIM |  |
| Neurological Disease | | | | | enlargement of axons | | | | | 2.54E-03 | | | | | CTSV |  |
| Neurological Disease | | | | | neurodegeneration of thalamus | | | | | 4.22E-03 | | | | | VIM |  |
| Neurological Disease | | | | | reactive astrogliosis | | | | | 5.91E-03 | | | | | CTSV |  |
| Neurological Disease | | | | | dystrophy of neurites | | | | | 6.75E-03 | | | | | VIM |  |
| Neurological Disease | | | | | cerebral amyloid angiopathy | | | | | 1.35E-02 | | | | | VIM |  |
| Neurological Disease | | | | | Movement Disorders | | | | | 2.77E-02 | | | | | CTSV,PPIA,TXN,VIM |  |
| Neurological Disease | | | | | neurodegeneration of hippocampus | | | | | 3.00E-02 | | | | | VIM |  |
| Neurological Disease | | | | | neurodegeneration of Purkinje cells | | | | | 3.41E-02 | | | | | CTSV |  |
| Neurological Disease | | | | | disorder of basal ganglia | | | | | 4.96E-02 | | | | | PPIA,TXN,VIM |  |

**Table E5: Top 5 cellular functions predicted by IPA**

| **IPA Cellular function** | **(p-value)** | **Molecules** |
| --- | --- | --- |
| **Cellular movement** | **3.05E-04 - 4.96E-02** | **↑ANXA3, ↑CFL1, ↑CSTV, ↓PPIA, ↓VIM, ↑TXN, ↑LY6D** |
| **Cellular development** | **3.94E-04 - 4.47E-02** | **↑CFL1, ↑CSTV, ↓PPIA, ↓VIM, ↑TXN, ↑RPL22, ↓FLG** |
| **Cell growth & proliferation** | **3.94E-04 - 4.47E-02** | **↑CFL1, ↑CSTV, ↓PPIA, ↓VIM, ↑TXN, ↓RPL22** |
| **Cell death & survival** | **8.36E-04 - 4.96E-02** | **↑TRIM29, ↑CSTV, ↓PPIA, ↓VIM, ↑TXN, ↓CCT6A** |
| **Cell morphology** | **8.46E-04 - 4.23E-02** | **↑CFL1, ↓VIM, ↑TXN, ↑CSTV** |

Ingenuity pathway analysis (IPA)

**Table E6: Top 5 canonical pathways predicted by IPA**

| **IPA Canonical pathways** | **(p-value)** | **Ratio** | **Molecules** |
| --- | --- | --- | --- |
| EIF2 Signaling | 1.51E-05 | 2.20E-02 | ↑RPL4, ↑RPS27, ↑RPL22, ↓RPS5 |
| Thioredoxin Pathway | 5.07E-03 | 1.67E-01 | ↑TXN |
| Regulation of eIF4 and p70S6K Signaling | 6.64E-03 | 1.40E-02 | ↑RPS27, ↓RPS5 |
| ILK Signaling | 1.06E-02 | 1.10E-02 | ↑CFL1, ↓VIM |
| mTOR Signaling | 1.08E-02 | 1.10E-02 | ↑RPS27, ↓RPS5 |

Ingenuity pathway analysis (IPA)

**Table E7: Summary of *FLG* genotype**

| **Donor ID** | **FLG genotype** | | **Biopsy site** |
| --- | --- | --- | --- |
| 1 | WT | WT | Normal |
| 2 | WT | WT | Normal |
| 3 | WT | WT | Normal |
| 4 | WT | WT | Normal |
| 5 | WT | WT | Normal |
| 6 | WT | WT | Normal |
| 7 | WT | WT | Normal |
| 8 | WT | WT | Normal |
| *9 | 2282del4 | WT | Normal |
| 10 | WT | WT | INV AE |
| 11 | WT | WT | INV AE |
| 12 | WT | WT | UN-INV & INV AE |
| 13 | WT | WT | INV AE |
| 14 | WT | WT | INV AE |
| 15 | WT | WT | UN-INV & INV AE |
| 16 | WT | WT | UN INV AE |
| 17 | R501X | WT | UN-INV & INV AE |
| 18 | WT | WT | INV AE |
| 19 | 2282del4 | WT | INV AE |
| 20 | R501X | WT | UN-INV & INV AE |
| 21 | R3419X | WT | UN-INV & INV AE |
| 22 | WT | WT | INV AE |
| 23 | WT | WT | INV AE |
| 24 | WT | WT | INV AE |
| 25 | R501X | R501X | INV AE |

Uninvolved (UN-INV), Involved atopic eczema (INV AE), WT (wild type)
*9 Control subject was excluded from IMF analysis as sequencing revealed a heterozygous *FLG* mutation
