# Supplementary material for: Proteomic analysis of filaggrin deficiency identifies molecular signatures characteristic of atopic eczema
Source: J Allergy Clin Immunol. 2017 Nov;140(5):1299–309. doi: 10.1016/j.jaci.2017.01.039 (PMC5667587; doi:10.1016/j.jaci.2017.01.039)
Supplement: Legends for Figures E1-E6 [file mmc3.docx]

**Figure E1 –** **LSE model is fully differentiated and displays filaggrin processing and maximal expression at 14 days.** LSE models were grown for up to 14 days at the air liquid interphase and the expression of filaggrin analysed by western blot at day 7, 9, 12 and 14 of culture (D7-D14). (N=3).

**Figure E2 –** **Filaggrin knockdown LSEs have a thicker epidermis.** shNT and shFLG LSE models were grown for 14 days and histology determined by h&E staining (figure 1). Thickness of the viable cell layer and stratum corneum was calculated using Image J software. Scatter plots of LOG­_10_ transformed data, mean ± 95% CIs (N=10, 5 measurements per donor).

**Figure E3 –** **Filaggrin knockdown confirmation in each of the 10 LSE donors used in the proteomics analysis.** shNT and shFLG LSE models were grown for 14 days and filaggrin expression and knockdown confirmed by immunoblotting. Solid line dictates that the samples were run on the same gel but in a discontinuous manner.

**Figure E4 - Principal component analysis of paired (shNT and shFLG) samples from 10 biological donors.** Each colour represents an individual biological donor.

**Figure E5 – Heat map analysis of paired (shNT and shFLG) samples from 10 biological donors.** Heat map depicting the protein expression changes between shNT and shFLG LSEs in each pair of LSEs derived from independent donors (N=10). Red/blue represents increased/decreased expression in the shFLG condition relative to shNT. Dendrogram highlights clustering between proteins.

**Figure E6 –** **Network linking the 17 differentially expressed proteins.** Differentially expressed proteins are highlighted in red (upregulated) and green (downregulated). The magnitude of response is dictated by the colour intensity. Proteins highlighted in pink have known roles in inflammatory skin disease.
